# Stargardt disease-associated missense and synonymous *ABCA4* variants result in aberrant splicing

**DOI:** 10.1093/hmg/ddad129

**Published:** 2023-08-09

**Authors:** Melita Kaltak, Zelia Corradi, Rob W J Collin, Jim Swildens, Frans P M Cremers

**Affiliations:** Department of Human Genetics, Radboud University Medical Center, Nijmegen, 6525 GA, The Netherlands; R&D Department, ProQR Therapeutics, Leiden, 2333 CK, The Netherlands; Department of Human Genetics, Radboud University Medical Center, Nijmegen, 6525 GA, The Netherlands; Department of Human Genetics, Radboud University Medical Center, Nijmegen, 6525 GA, The Netherlands; R&D Department, ProQR Therapeutics, Leiden, 2333 CK, The Netherlands; Department of Human Genetics, Radboud University Medical Center, Nijmegen, 6525 GA, The Netherlands

## Abstract

Missense variants in *ABCA4* constitute ~50% of causal variants in Stargardt disease (STGD1). Their pathogenicity is attributed to their direct effect on protein function, whilst their potential impact on pre-mRNA splicing disruption remains poorly understood. Interestingly, synonymous *ABCA4* variants have previously been classified as ‘severe’ variants based on *in silico* analyses. Here, we systemically investigated the role of synonymous and missense variants in *ABCA4* splicing by combining computational predictions and experimental assays. To identify variants of interest, we used SpliceAI to ascribe defective splice predictions on a dataset of 5579 biallelic STGD1 probands. We selected those variants with predicted delta scores for acceptor/donor gain > 0.20, and no previous reports on their effect on splicing. Fifteen *ABCA4* variants were selected, 4 of which were predicted to create a new splice acceptor site and 11 to create a new splice donor site. In addition, three variants of interest with delta scores < 0.20 were included. The variants were introduced in wild-type midigenes that contained 4–12 kb of *ABCA4* genomic sequence, which were subsequently expressed in HEK293T cells. By using RT-PCR and Sanger sequencing, we identified splice aberrations for 16 of 18 analyzed variants. SpliceAI correctly predicted the outcomes for 15 out of 18 variants, illustrating its reliability in predicting the impact of coding *ABCA4* variants on splicing. Our findings highlight a causal role for coding *ABCA4* variants in splicing aberrations, improving the severity assessment of missense and synonymous *ABCA4* variants, and guiding to new treatment strategies for STGD1.

## Introduction

Biallelic *ABCA4* variants impair the function of the ATP-binding cassette subfamily A member 4 protein (ABCA4) and are responsible for Stargardt disease type 1 (STGD1) and related retinopathies (1). Almost 2400 distinct disease-associated variants in the *ABCA4* gene have been discovered (www.lovd.nl/ABCA4). Located in the rod photoreceptor disc membranes and the cone photoreceptor cell membrane, ABCA4 acts as a flippase of *N*-retinylidene-phosphatidylethanolamine (*N*-Ret-PE), the Schiff-base adduct of retinal and phosphatidylethanolamine formed during the visual cycle (2). Its disfunction leads to accumulation of di-retinoid compounds in photoreceptor cells with a consequent visual impairment with varying degrees of severity (3–6). STGD1 probands display very different phenotypes based on the allele combinations, from early-onset STGD1 or panretinal cone-rod dystrophy because of two severe alleles, to late-onset STGD1 characterized by foveal sparing because of p.(Asn1868Ile) or p.(Gly1961Glu) in *trans* with a severe allele (7). Cases of low penetrant alleles, the discovery of sex imbalance for some combinations of *ABCA4* variants, and putative *trans*-modifiers in *PRPH2* and *ROM1* strongly suggest a multifactorial or polygenic inheritance of disease for a subset of STGD1 cases (8–10). Therefore, to provide a prognosis of disease progression as accurately as possible it is important to assess the severity of *ABCA4* variants.

Missense variants in *ABCA4* are the major underlying cause for STGD1, accounting for 48% of all unique and 62% of all disease-associated variants in STGD1 probands (11). Comprehensive *in vitro* functional analyses of missense variants determined their pathogenic effect across all structural domains in ABCA4, where most of the variants were predicted to either impair protein folding and trafficking or severely reduce its ATPase activity (12–16). So far, the effect of missense variants in *ABCA4* was determined solely on the change in the amino acid sequence, whilst evidence of their possible effect on splicing is lacking. Based on extensive *in silico* studies by Cornelis *et al*. (11), the severity prediction of *ABCA4* variants was carried out for variants identified in 5579 biallelic STGD1 probands. Subsequently, seven synonymous variants were classified as ‘severe’ or ‘moderately severe,’ without experimental proof. This observation led to the hypothesis that disease-associated coding variants in *ABCA4*, either non-synonymous or synonymous, might have disruptive effects on splicing.

In this study, we report causality for synonymous and missense variants in *ABCA4* by attributing them novel missplicing events. By combining the splice prediction tool SpliceAI (17) and *in vitro* midigene assays, we were able to shed additional light on the pathogenicity of previously classified severe STGD1 variants and to attribute new severity scores to others. Our findings have significant implications for a more accurate prognosis of disease progression and the development of appropriate therapeutic strategies targeting the (splice) coding *ABCA4* variants in individuals with STGD1.

## Results

### Selection of coding variants in *ABCA4* based on SpliceAI predictions

We set out to identify missense and synonymous variants in *ABCA4* that might affect splicing by assigning SpliceAI delta scores to all variants previously identified by Cornelis *et al*. (11). Specifically, this study assessed all *ABCA4* variants detected in 5579 biallelic STGD1 probands and categorized them based on their severity. Variants that were of particular interest were those classified as moderately severe or severe, as their effect may not solely be based on an amino acid change. Also, missense variants that were previously classified as variants of uncertain significance and those that could not be categorized because of limited data were taken along with the intent of assigning them a severity score based on their impact on splicing (11). Importantly, we omitted the variants located at the first, second, penultimate and last position within exons under the assumption that these are very likely to affect splicing.

The computational analysis prioritized those variants that displayed a delta score (DS) > 0.20 for splice acceptor gain (AG) or donor gain (DG). This threshold, regarded as the ‘high recall’ threshold by the developers (17), showed reliability in previous computational analyses of *ABCA4* deep-intronic and non-canonical splice site variants (18). Here, we hypothesized that DS > 0.20 likely predicts the formation of new splice sites caused by coding missense and synonymous *ABCA4* variants, which will compete with the canonical splice acceptor site (SAS) or splice donor site (SDS). As a result, 15 variants of interest were identified, with 4 variants having an AG DS > 0.20 and 11 with a DG DS > 0.20. Among these variants, only c.3407G>T was attributed both a DG DS and an AG DS > 0.20. Since the DG DS was higher than AG DS (0.95 > 0.30), this variant was taken into the DG variants group. We added three other variants to the analysis even though their AG DSs were < 0.20. Two of these variants are located nearby a so-called dual SAS/SDS in exon 30 (c.4454C>T and c.4457C>T), and thus are likely to affect the splicing. Additionally, one variant, c.4203C>A, was previously classified as benign based on ACMG/AMP criteria and served as a negative control for the splicing analysis (19). The chosen AG and DG variants are presented in Tables 1 and 2, respectively, while the complete overview of attributed SpliceAI predictions is shown in Supplementary Material, Table S1.

**Table 1:** *ABCA4* coding variants predicted to introduce new SASs. DS AG, delta score for strongest acceptor gain prediction. Severity category assessed by Cornelis *et al.* (11). N/A, not applicable because of limited amount of data in previous study

cDNA variant	Protein variant	Severity category^11^	DS AG
c.1977G>A	p.(Met659Ile)	Severe	0.99
c.3703A>G	p.(Asn1235Asp)	Mild/Moderate	0.97
c.5088C>G	p.(Ser1696Arg)	Causative variant of unknown severity	0.34
c.5367C>G	p.(Ser1789Arg)	N/A	0.32
c.4203C>A	p.(Pro1401=)	Benign	0.17
c.4454C>T	p.(Ser1485Leu)	N/A	0.14
c.4457C>T	p.(Pro1486Leu)	Moderate	0.12

**Table 2:** *ABCA4* coding variants predicted to introduce new SDSs. DS DG, delta score for strongest donor gain prediction. Severity category assessed by Cornelis *et al.* (11). N/A, not applicable because of limited amount of data in previous study

cDNA variant	Protein variant	Severity category^11^	DS DG
c.6207C>T	p.(Gly2069=)	N/A	0.97
c.3407G>T	p.(Gly1136Val)	Severe	0.95
c.2128A>G	p.(Met710Val)	N/A	0.91
c.4446C>A	p.(Val1482=)	N/A	0.89
c.3462C>T	p.(Gly1154=)	Moderately severe/Severe	0.88
c.6272T>A	p.(Leu2091Gln)	N/A	0.86
c.2273C>T	p.(Ala758Val)	N/A	0.84
c.4469G>A	p.(Cys1490Tyr)	Severe	0.80
c.6339C>G	p.(Ile2113Met)	Causative variant of unknown severity	0.78
c.1022A>T	p.(Glu341Val)	N/A	0.65
c.3096A>T	p.(Gly1032=)	N/A	0.60

### *ABCA4* variants leading to alternative splice acceptor sites

**Figure 1:**
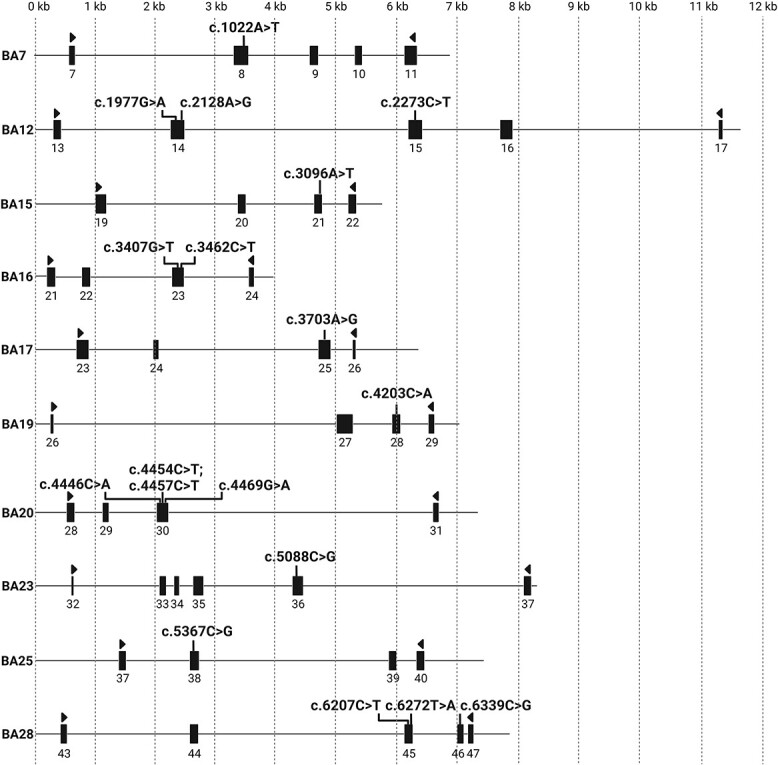
Schematic representation of wild-type *ABCA4* midigene-constructs used in this study for introducing *ABCA4* coding variants for splicing analysis. The numbers below rectangles indicate the *ABCA4* exons. The binding locations of primers used for transcript analysis by RT-PCR are represented by triangles.

Seven variants were selected based on their AG DS and introduced into previously described wild-type (WT) BA constructs, ensuring that the exon containing the variant was flanked by at least one exon and the adjacent intronic sequences (20). The details of the used WT constructs can be found in Figure 1 and Supplementary Material, Table S2. Despite the use of a high-fidelity DNA polymerase, we observed polymorphisms (Supplementary Material, Table S3) introduced upon mutagenesis. However, they are not predicted to alter the pre-mRNA splicing.

**Figure 2:**
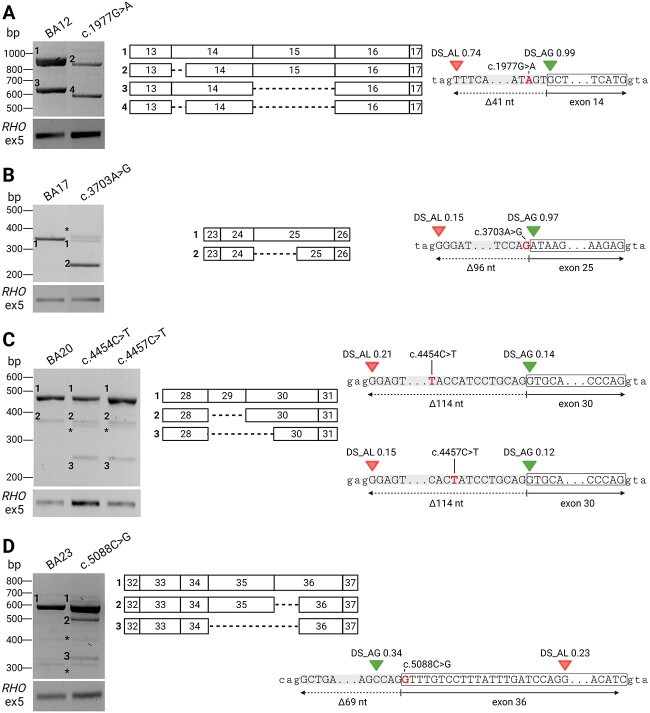
Splicing defects caused by five missense *ABCA4* variants creating new SASs. The RT-PCR of RNA derived from mutant midigenes and the corresponding WT plasmids were visualized with gel electrophoresis. The green triangles represent the splice site predictions for acceptor gain (AG) by SpliceAI. Acceptor losses (AL; red triangles) were also shown if the delta scores (DSs) were > 0.10. Fragments for which the sequence information suggested a PCR artifact are indicated by asterisks. (A) For the mutant c.1977G>A midigene, two splicing defects were detected (fragments 2 and 4) that corresponded to a skip of the first 41 nt of exon 14 and a complex splice defect resulting in the absence of the first 41 nt of exon 14 and the complete exon 15. Skipping of exon 15 was also observed in the WT construct as seen previously (20). (B) RT-PCR and sanger sequencing of the c.3703A>G BA17 midigene revealed an in-frame 96-bp deletion (fragment 2) at the 5′ end of exon 25, as opposed to the WT BA17 which did not show this defect. (C) Missense variants c.4454C>T and c.4457C>T led to the generation of fragments that were confirmed by Sanger sequencing to lack exon 29 (fragment 2) completely, and a combination of a deletion of exon 29 and the first 114 nt of exon 30 (fragment 3). The latter was absent in the RNA derived from WT BA20. (D) Variant 5088C>G led to a novel transcript with an in-frame deletion of 69 nt at the 5′ end of exon 36 (fragment 2). A complete deletion of exon 35 was observed in fragment 3.

As depicted in Figure 2, nearly all the investigated coding variants led to expected misspliced *ABCA4* RNAs, apart from c.4203C>A and c.5367C>G that did not alter the splicing when compared with the corresponding WT control (Supplementary Material, Figs S1 and S2, respectively). Variant c.1977G>A was predicted to severely affect the SAS of exon 14. In fact, we identified the expected isoform with an alternative SAS 41 nt downstream of the original SAS, together with the *ABCA4* transcript lacking both the 41 nt and the complete exon 15. These events led to predicted premature stop codons, resulting in p.Phe647Alafs*105 and p.Phe647Alafs*73, respectively. No WT *ABCA4* mRNA was identified for this variant (Fig. 2A and Supplementary Material, Fig. S3).

Variant c.3703A>G led to a transcript missing 96 nt at the 5′ end of exon 25, as predicted by SpliceAI. The WT isoform was the only one identified in the WT control plasmid, as opposed to the c.3096A>G sample where this was present only in traces (Figs 2B and Supplementary Material, Fig. S4).

Exon 30 variants c.4454C>T and c.4457C>T weakened the predicted strength of the canonical SAS of exon 30 and generated an alternative SAS 114 nt downstream of the canonical SAS. This aberration was found exclusively in combination with the complete skip of exon 29. All samples displayed the WT RNA as the most predominant isoform, and small amounts of an *ABCA4* transcript lacking exon 29 (Fig. 2C and Supplementary Material, Fig. S5).

Variant c.5088C>G was anticipated to impose the formation of a new SAS 66 nt downstream from the original SAS. However, the isoform identified upon RT-PCR, as shown in Figure 2D and Supplementary Material, Figure S6, lacked 69 nt from the 5′ end of exon 36. This frameshift event was observed in only 15.0 ± 0.5% compared with total *ABCA4*. The WT *ABCA4* transcript and a transcript lacking exon 35 and the first 69 nt of exon 36 were detected in both mutant and WT midigenes.

### *ABCA4* variants resulting in alternative splice donor sites

The 11 *ABCA4* variants that were predicted to create a new SDS and thus compete with the canonical SDS, were introduced into the appropriate WT constructs (Fig. 1 and Supplementary Material, Table S2) (20).

**Figure 3:**
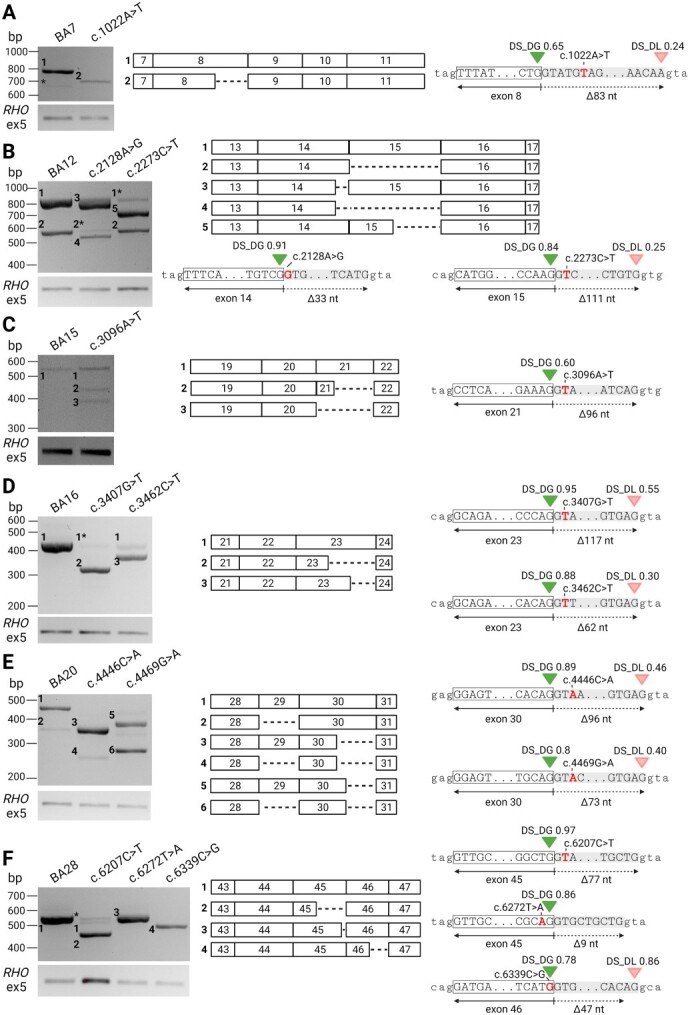
Splicing defects caused by 11 variants creating new SDSs. The RNA products content, extracted from midigene-transfected HEK293T cells, were studied with RT-PCR and Sanger sequencing. The green triangles represent the splice site predictions for donor gain (DG) by SpliceAI. Donor losses (DL; red triangles) were also shown if the delta scores (DSs) were > 0.10. (A) Variant c.1022A>T led to the formation of an alternative SDS 83 nt upstream of the canonical SDS. (B) Variants c.2128A>G and c.2273C>T caused in-frame deletions at the 3′ end of exons 14 and 15, respectively, because of newly created SDS upstream the canonical donor sites. *ABCA4* Δexon 15 was observed in both mutant and WT constructs as a single event (fragment 2) or, in case of c.2128A>G, in combination with the predicted splicing aberration (fragment 4). (C) RT-PCR for the c.3096A>T BA15 construct identified two isoforms that were not present in the BA15 WT sample (fragments 2 and 3). Fragment 2 missed the last 96 nt of exon 21, which was predicted by the *in silico* analysis. Asterisks denote fragments in which the sequence information resulted from PCR artifacts, or the sequence could not be identified. (D) Variants c.3407G>T and c.3462C>T caused the activation of cryptic SDS 117 nt (fragment 2) and 62 nt (fragment 3) upstream the canonical SDS in exon 23, respectively. (E) Variants c.4446C>A and c.4469G>A resulted exclusively in misspliced *ABCA4* RNAs. For c.4446C>A, sequence analysis confirmed fragment 3 to lack 96 nt at the 3′ of exon 30, while fragment 4 missed the complete exon 29 and first 96 nt of exon 30. Similarly, for c.4469G>A, fragments 5 and 6 represented the Δexon 29 and the latter in combination with a deletion of 73 nt at the 3′ of exon 30. Complete skip of exon 29 was detected upon expression of the BA16 WT, which was observed for variant c.4446C>A as well. (F) Aberrant splicing was detected for variants c.6207C>T, c.6272T>A and c.6339C>G introduced in BA28, where new cryptic SDSs were activated 77 nt (fragment 2) or 9 nt (fragment 3) upstream of the canonical SDS of exon 45, or 47 nt upstream the canonical SDS of exon 46 (fragment 4).

Variant c.1022A>T, as predicted by SpliceAI, resulted in the deletion of the last 83 nt of exon 8 (fragment 2 in Fig. 3A and Supplementary Material, Fig. S7). This resulted in a frameshift and premature stop codon (p.Tyr339Cysfs*37). This event was not identified in the WT BA7 sample.

The high DSs for the BA12 DG variants c.2128A>G and c.2273C>T predicted the creation of new SDSs, competing with the canonical SDSs of exons 14 and 15, respectively. These in-frame truncations were identified with RT-PCR shown in Figure 3B and Supplementary Material, Figure S8, and corresponded with fragment 3 (loss of last 33 nt of exon 14, p.Met710_Met720del) and fragment 5 (loss of last 111 nt of exon 15, p.Ala758_Val794del). In addition, skipping of *ABCA4* exon 15 was identified in all mutant midigenes and the control WT sample (20), either as a single event (fragment 2, p.His721_Val794del), or combined with the predicted SDS disruption (fragment 4, p.Met710_Val794del).

For the mutant c.3096A>T BA15 construct, the RT-PCR analysis revealed the presence of two shortened *ABCA4* RNA products, one missing the last 96 nt of exon 21 (fragment 2, p.Gly1032_Ser1063del), and the other one lacking the complete exon 21 (fragment 3, p.His1017Glnfs*111) (Fig. 3C and Supplementary Material, Fig. S9). The creation of the alternative SDS in exon 21 (fragment 2) was strongly predicted by SpliceAI. The WT BA15 sample yielded only the WT *ABCA4* transcript (Fig. 3C).

The RT-PCR of samples transfected with c.3407G>T and c.3462C>T BA16 midigenes confirmed the previously assigned (11) severe nature of the two investigated variants, represented in Figure 3D and Supplementary Material, Fig. S10. In fact, the presence of both variants severely affected the abundance of WT *ABCA4*, which was present at very low levels when compared with the WT midigene. Variant c.3407G>T led to the expression of an *ABCA4* RNA product missing 117 nt at the 3′ end of exon 23. This event was strongly predicted by SpliceAI, where the canonical SDS was weakened and an alternative SDS was favored. This resulted in an in-frame deletion of 39 amino acids (p.Gly1136_Glu1174del). Similarly, c.3462C>T created an alternative SDS 62 nt upstream the canonical exon 23 SDS, also strongly predicted by SpliceAI. The open reading frame was disrupted (p.Leu1155Aspfs*19). None of the alternative isoforms were detected in the BA16 WT sample.

As shown in Figure 3E and Supplementary Material, Fig. S11, exon 30 variants c.4446C>A and c.4469G>A resulted in complex aberrant splicing events that involved exons 29 and 30. SpliceAI predicted that c.4446C>A would lead to the use of a cryptic SDS located 96 nt upstream the canonical SDS of exon 30. This RNA product was present at 92.7 ± 0.1% when compared with total *ABCA4* RNA, together with the isoform lacking a combination of the upper event with *ABCA4* Δexon 29 present at 7.3 ± 0.1%. The predicted ABCA4 proteins are p.Val1482_Gln1513del and p.[Ser1418_Pro1451delinsArg,Val1482_Gln1513del], but as the latter was present in < 15% of total *ABCA4* RNA, it was not represented in the new protein notation. As for the c.4469G>A variant, two similarly expressed transcripts were identified with RT-PCR. The first showed a 73-nt shortened exon 30 at the 3′ end, which is in line with the previously determined splicing prediction and previous research (21). This led to a shift in the open reading frame and a premature stop codon (p.Cys1490Glufs*12). In addition, the second identified fragment lacked the complete exon 29, whereas exon 30 underwent a 2-nt elongation at its 5′ end and the abovementioned 73-nt truncation at its 3′ end.

The *ABCA4* variants c.6207C>T, c.6272T>A and c.6339C>G were predicted to strengthen cryptic SDSs in exons 45 and 46, which was confirmed by RT-PCR results presented in Figures 3F and Supplementary Material, Fig. S12. Specifically, c.6207C>T caused the activation of a new cryptic SDS that excluded the last 77 nt of exon 45, resulting in p.Thr2070*. Variant c.6272T>A produced an in-frame deletion of the last 9 nt in exon 45 because of a new SDS upstream of the canonical SDS, leading to p.Val2092_Leu2094del. Variant c.6339C>G in exon 46 caused a shift in the open reading frame by activating a new SDS 47 nt upstream the 3′ end of exon 46, resulting in p.Val2114Hisfs*5. Unlike the BA28 WT, none of the three mutant BA28 constructs produced WT RNA (apart from c.6207C>T for which WT RNA was measured at non-significant levels, 3.5 ± 0.7%).

### Severity categorization of missense variants based on the levels of correctly spliced RNA

**Figure 4:**
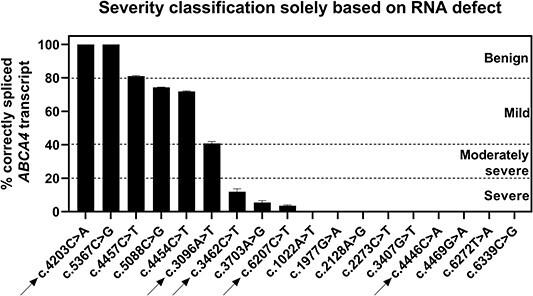
Percentages of correctly spliced *ABCA4* mRNA. The intervals of the severity categories are as follows: 0% < Severe ≤ 20%, 20% < Moderately severe ≤ 40%, 40% < Mild ≤ 80%, 80% < Benign ≤ 100%. Data are shown as mean ± SEM. The arrows point to the synonymous variants.

To assess the severity of variants based on their impact on splicing, cDNA products were quantified using the gel images. Details regarding the analysis for AG and DG variants can be found in Supplementary Material, Tables S4 and S5, respectively. Figure 4 illustrates the percentages of WT RNA together with the severity thresholds estimated earlier (11). As the c.4203C>A and c.5367C>G variants did not obstruct the splicing at all, these were classified as ‘benign.’ Variants c.5088C>G, c.4454C>T and c.3096A>T were categorized as ‘mild,’ as they yielded between 40% and 80% of WT RNA. None of the variants were considered ‘moderately severe,’ whereas the remaining 12 variants were labeled as ‘severe’ since they resulted in < 20% of WT *ABCA4*. The intervals for mild, moderately severe and severe *ABCA4* alleles were derived from unpublished theoretical modelling studies in our group. The overview of all splicing events was summarized in Supplementary Material, Figure S13. Since several variants led to in-frame deletions within the coding sequence of *ABCA4*, there is, theoretically, a probability of remaining residual activity of ABCA4. Therefore, we performed an *in silico* analysis where we linked these deletions to the amino acid sequence and determined their possible impact on the protein’s function. As expected, all of the in-frame deletions resulted in removal of critical elements of ABCA4, without which the protein would not be able to maintain its functionality (Supplementary Material, Tables S4 and S5).

In addition, the severity of the missense variants was assessed by consulting data from previously published STGD1 probands for their age of disease onset and the *ABCA4* allele found in *trans*. A missense variant would be considered of severe nature if: 1. the *ABCA4* variant in *trans* was previously categorized as ‘mild,’ it must be accompanied by a second severe allele in order for STGD1 to manifest, or 2. the age of onset was before 10 years of age, which in most cases is associated with the presence of two severe *ABCA4* alleles. The details of this analysis are reported in Supplementary Material, Table S6. For the 18 investigated variants, we were able to attribute the ‘severe’ label to six variants, i.e. c.1977G>A, c.3407G>T, c.4469G>A, c.6207C>T, c.6272T>A and c.6339C>G. Interestingly, all mentioned variants yielded ≤ 20% of WT RNA and were, thus, classified as ‘severe’ in Figure 4, which is in line with the above *in silico* analysis.

Table 3 provides a summary of the newly discovered impact of the investigated variants on RNA and protein. The updated RNA and protein products have been deposited into the LOVD database for *ABCA4* (www.lovd.nl/ABCA4). Moreover, the table reports their ACMG/AMP classification according to the previously published studies (22), as well as their updated ACMG/AMP classification, taking into account the impact on splicing reported in this study. It is important to note that, out of 18 investigated missense variants, the ACMG/AMP classification of four variants was raised to a higher severity class, indicating increased reliability of their pathogenic nature.

**Table 3:** List of coding *ABCA4* variants and their novel effect on RNA and protein, with the old ACMG/AMP classification assessed by Cornelis et al. (22) and the updated ACMG/AMP classification based on the observed splice defects. The variants for which the ACMG/AMP classification changed are represented in bold. VUS, variant of uncertain significance

**DNA variant**	**RNA variant**	**Protein variant**	**% correctly spliced RNA**	**Previous ACMG/AMP classification of coding variant** ^**22**^	**Severity classification based on splice assays in study**	**Combined ACMG/AMP classification in this study**
c.1022A>T	r.1017_1099del	p.Tyr339Cysfs*37	0	Likely pathogenic	Severe	Likely pathogenic
**c.1977G>A**	**r.[1938_1978del,2161_2382del]**	p.[Phe647Alafs*73,Phe647Alafs*105]	**0**	**VUS**	**Severe**	**Likely pathogenic**
c.2128A>G	r.[2128_2160del,2128_2382del]	p.[Met710_Met720del,Met710_Val794del]	0	VUS	Severe	VUS
c.2273C>T	r.[2128_2382del,2272_2382del]	p.[Ala758_Val794del,His721_Val794del]	0	VUS	Severe	VUS
c.3096A>T	r.[3096a>u,3051_3190del,3095_3190del]	p.[Gly1032=,His1017Glnfs*111,Gly1032_Ser1063del]	40.8	VUS	Mild	VUS
**c.3407G>T**	**r.3406_3522del**	**p.Gly1136_Glu1174del**	**0**	**VUS**	**Severe**	**Likely pathogenic**
**c.3462C>T**	**r.3461_3522del**	p.Leu1155Aspfs*19	**12.0**	**VUS**	**Severe**	**Likely pathogenic**
**c.3703A>G**	**r.3608_3703del**	**p.Gly1203_Asn1235delinsAsp**	**5.5**	**VUS**	**Severe**	**Likely pathogenic**
c.4203C>A	r.4203c>a	p.(Pro1401=)	100	Benign	Benign	Benign
c.4446C>A	r.4444_4539del	p.Val1482_Gln1513del	0	VUS	Severe	VUS
c.4454C>T	r.[4454c>u,4254_4466del]	p.[Ser1485Leu,Ser1418_Cys1488delinsArg]	71.9	VUS	Mild	VUS
c.4457C>T	r.4457c>u	p.(Pro1486Leu)	81.1	Pathogenic	Benign	Pathogenic
c.4469G>A	r.[4254_4352delinsag,4467_4539del]	p.[Ser1418Argfs*80,Cys1490Glufs*12]	0	Pathogenic	Severe	Pathogenic
c.5088C>G	r.[5088c>g,5019_5087del]	p.[Ser1696Arg,Leu1674_Ser1696del]	74.3	Likely pathogenic	Mild	Likely pathogenic
c.5367C>G	r.5367c>g	p.(Ser1789Arg)	100	VUS	Benign	VUS
c.6207C>T	r.6206_6282del	p.Thr2070*	3.5	VUS	Severe	VUS
c.6272T>A	r.6274_6282del	p.Val2092_Leu2094del	0	VUS	Severe	VUS
c.6339C>G	r.6340_6386del	p.Val2114Hisfs*5	0	Likely pathogenic	Severe	Likely pathogenic

## Discussion

In this study, we investigated whether synonymous and missense coding variants in *ABCA4* had an effect on pre-mRNA splicing. Using SpliceAI to predict the possible effect on splicing and midigene-based splice assays to validate the predictions, we report novel missplicing events in 16 out of 18 investigated coding *ABCA4* variants. These novel aberrations in splicing led to in-frame deletions within exons or frameshifts, because of newly activated (one SAS, five SDS) or strengthened (four SAS, five SDS) cryptic splice sites. Interestingly, *in silico* analysis of in-frame deletions reported in Supplementary Material, Tables S4 and S5 suggests that although these are not obstructing the open reading frame, they affect major domains within the ABCA4 protein and are therefore having a severe effect on the protein’s remaining activity. Furthermore, 12 variants led to ≤ 20% of WT *ABCA4* RNA, which corroborate the certainty regarding their pathogenicity and their classification as ‘severe’ STGD1 variants.

So far, therapeutic strategies targeting missense variants associated with inherited retinal diseases (IRDs) focused on genetic-based approaches in order to express the wild-type protein, which involved gene augmentation, nuclease-based genome editing and RNA editing. A potential RNA therapy currently applicable to missense variants is ADAR-mediated editing, that targets, however, only missense G>A variations. This therapeutic strategy employs short RNA molecules that recruit the naturally occurring endogenous adenosine deaminase acting on RNA (ADAR) enzymes able to convert an adenosine to inosine, recognized as guanine (23–25). It is estimated that 23% of all missense variants underlying IRDs are treatable by ADAR-mediated editing, while this number falls to 10% for all causal *ABCA4* variants (26). Next to this, Ascidian Therapeutics showed promising results with their Exon Editor technology, which makes use of the RNA trans-splicing process to replace the disease-associated pre-mRNA sequence in *ABCA4*. This approach would address both the splicing aberration and the amino acid change upon the missense variants in this study (27). On the other hand, encouraging progress in the development of potential therapeutic modalities targeting splicing variants in *ABCA4* has been reported by several groups, either by applying permanent intronic DNA-based alterations (28) or reversible RNA-based approaches that involve the application of antisense oligonucleotides (29–35). The variants reported in this study could make a great target for AON-based approaches, which would solve their pathogenicity imposed by the aberration in splicing. However, the presence of the amino acid change would still hinder the protein’s full functionality. Therefore, it is important to determine the effect of the amino acid substitution to define its effect over the overall protein’s functionality. The specialized function of ABCA4 imposes challenges in determining its activity as it is not behaving like many other transmembrane proteins. While its structure resembles the one of the transmembrane conductance regulator (CFTR) from the ABCC7 group (36), ABCA4’s function is more complex than CFTR’s straightforward ion flux, which allowed setting up protocols for *in vitro* functional assays (37). So far, functional analyses of ABCA4 have been performed only in extracellular environments or cultured cells and in conditions that involve the use of mild detergents, which can, regardless of their mild nature, affect the activity of the protein and give a false image of one variant’s effect. For example, the functional assessment of the very common p.(Gly1961Glu) variant suggests the variant’s detrimental effect on ABCA4 function, regardless of its previously established mild or hypomorphic nature (15). Considering all, the above-mentioned ADAR-mediated editing and Exon Editor technology would address the entire underlying cause, whereas AONs would only address the aberrant splicing, leaving the impact of the amino acid change untreated. However, since the ADAR system relies firstly on the recruitment and, secondly, the limited availability of endogenously expressed ADARs, correcting the target can only be achieved within the confines of the available ADAR enzymes.

We observed very low or no expression of correctly spliced *ABCA4* mRNA for 12 out of 18 tested variants, and the severity category for some of them worsened. In fact, with regard to the ACMG/AMP classification, the certainty for severity increased in four variants because of the observed in-frame deletions in the RNA or shifts in the open reading frame. Before this study, variants c.1977G>A, c.3407G>T, c.3462C>T and c.3703A>G were classified as variants of uncertain significance. Based on the splice assay results they became likely pathogenic. From the five synonymous variants that were part of the analysis, only the c.4203C>A variant, that was previously classified as ‘benign’ and thus served as a negative control (11), led to exclusively correctly spliced *ABCA4* transcript, as expected. On the other hand, three synonymous variants (c.3462C>T, c.4446C>A, c.6207C>T) yielded ≤ 20% of correctly spliced mRNA which allowed them to be labeled as ‘severe.’ In addition, the c.3096A>T variant led to the generation of 41% correctly spliced mRNA, which categorized it as a ‘mild’ STGD1 variant. Since these variants do not alter the amino acid sequence, the discovery of missplicing suggests that a splicing modulation approach could be the only necessary treatment in alleviating the STGD1 phenotype. In addition, this study excluded coding *ABCA4* variants located at the first, second, penultimate and last positions of exons as these very likely have an impact on splicing. The *in vitro* assessment of these would likely reveal additional missense and synonymous *ABCA4* variants that influence splicing and are therefore interesting candidates for future studies. Moreover, we omitted variants that were previously classified as ‘moderately severe’ and ‘mild,’ regardless of whether the DS for AG or DG attributed by SpliceAI exceeded the predefined threshold. These variants may have a partial effect on splicing based on their severity assessment, making them compelling candidates for future investigations.

Bioinformatic-based assessment of pathogenicity prediction is vastly used to determine the possible underlying cause for the association between variants and disease. However, it is worth mentioning that certain pathogenic events linked to missplicing arise in a tissue-specific environment, and *in silico* prediction tools that operate using generic splicing data may fail to predict them. Therefore, these pathogenic events may require validation through *in vitro* experiments (18). For example, the common severe *ABCA4* c.5461-10T>C variant is not predicted to cause splicing aberrations when using SpliceAI (delta scores for AG, AL, DG, and DL < 0.08); however, *in vitro* investigations clearly showed its severe effect because of exon 39 or exons 39/40 skipping resulting in frameshifts (38). The use of midigenes for splicing prediction purposes highly facilitated the discovery of novel splicing aberrations in *ABCA4* that allowed for development of splicing-targeted therapies. Results from previous investigation have reaffirmed the preference for midigenes over minigenes. Minigenes usually contain one exon and short sequences of adjacent introns, thus their very restricted genomic content may provide misleading results (20). Most variants examined in this study were attributed DS > 0.20 by SpliceAI either for creation or activation of alternative cryptic SASs or SDSs, which were confirmed, in most cases, by midigene assays. However, c.5088C>G was the only variant to cause the splicing aberration in sites predicted differently by SpliceAI, whilst c.5367C>G did not display any splicing disruption and yielded 100% correctly spliced *ABCA4* RNA. Variant c.5367C>G was attributed a DS above the 0.20 threshold (DS = 0.32) for AG and did not fall within any severity category because of limited data from previously screened STGD1 probands (11). In addition, this variant is also the only AG variant that did not display an AL DS > 0.10, as opposed to all other selected AG variants, which suggests that the AG prediction might need to be combined with a prediction for AL in order to result in the predicted missplicing event. It is likely that c.5367C>G could result in splicing aberration if investigated in a wider genomic context, or that its pathomechanism is yet to be discovered. In fact, some studies have highlighted inconsistencies between splicing aberrations detected using midigenes or simpler cellular models, as opposed to more sophisticated models derived from reprogrammed induced pluripotent stem cells, such as photoreceptor precursor cells or retinal organoids. As the genomic context of the complex models closely resembles that of the native retinal environment, these are more representative of the actual splicing process in the retina (29,32,39,40). These findings point to the limits imposed by the midigene system. Therefore, development of novel therapeutic compounds should always involve the validation in complex cellular systems, while midigenes could serve as a screening tool only.

Four exon 30 variants were analyzed, two of which, i.e. c.4454C>T and c.4457C>T, were associated with AG DSs of 0.14 and 0.12, while the other two variants, c.4446C>A and c.4469G>A, were attributed high DG DSs (0.89 and 0.80, respectively). We identified that variants c.4454C>T and c.4457C>T strengthened an existing cryptic SAS at position c.4466, while variant c.4469G>A strengthened an existing cryptic SDS at the neighboring c.4467 position. Moreover, variant c.4446C>A leads to activation of a second existing cryptic SDS in position c.4444. The existence of these ‘dual splice sites,’ a term coined for neighboring and partially overlapping acceptor and donor splice sites, may result in the binding of many splice factors and thereby could play a role in the effect of the relatively mild variants c.4454C>T and c.4457C>T (41). However, we also observed that all these variants contributed to changes of exonic splicing regulatory (ESR) sequences, as shown in Supplementary Material, Figure S14, regardless of the significant differences in the attributed DSs. In fact, both the low DS variants c.4454C>T and c.4454C>T contributed to the deletion of exonic splice enhancers (ESEs), just as the c.4446C>A variant. At the same time, variants c.4446C>A, c.4454C>T and c.4469G>A led to generation of novel exonic splice silencers in exon 30. These observations support the hypothesis initially proposed by Moles-Fernández and colleagues that SpliceAI may face challenges in detecting changes in the ESR landscape, thus overlooking the effect one variant may have on splicing (42). Previous research has shown that even synonymous coding variants can have a role in altering ESRs and causing missplicing events associated with disease. For example, Collin *et al*. described a synonymous variant in the *TECTA* gene to cause a loss of an ESE, resulting in defective splicing and, consequently, DFNA8/12 hearing impairment (43). Therefore, besides assessing the effect one variant may have on the splice sites, it is crucial to determine its effect on the ESR landscape to accurately predict potential splicing aberrations.

SpliceAI was deemed a dependable *in silico* tool for assessing potential splice alterations in non-canonical splice site and deep-intronic *ABCA4* variants, following a comparison of several established and deep learning tools (18). However, it is worth mentioning that positive predictions for these types of variants should not be generalized to the overall validity of variant interpretation, especially since the tool’s training may only focus on estimating the potential impact on splicing for such variants rather than the coding variants. Nonetheless, the fact that 15 out of 18 investigated variants demonstrated the missplicing predicted by SpliceAI in midigene-based assays, lends support to the reliability of this *in silico* tool for interpreting splicing in coding *ABCA4* variants. We observed a significant difference in the mean DSs between AG (0.44) and DG (0.83). Among the analyzed AG variants, two variants, c.1977G>A and c.3703A>G, were attributed considerably higher DSs of 0.99 and 0.97, respectively, followed by DS of 0.34 that was attributed to c.5088C>G. The large discrepancy in DS was reflected in the amount of correctly spliced RNA, as c.1977G>A and c.3703A>G yielded considerably lower amount of correctly spliced *ABCA4* and were categorized as ‘severe’ variants, unlike the other investigated AG variants, none of which fell within the ‘severe’ category. Furthermore, the majority of analyzed DG variants was assigned to the ‘severe’ category based on the amount of correctly spliced *ABCA4*, save the ‘mild’ c.3096A>T variant, which also had the lowest DS when compared with the rest of investigated DG variants. Even though the strength of a DS attributed by SpliceAI does not necessarily correlate with the amount of misspliced product, but rather represents the likelihood for the event to happen, we could not overlook this interesting observation. The possible correlation between the strength of the DS and the amount of correctly spliced RNA in missense *ABCA4* variants needs further investigation in order to draw more robust conclusions. In addition, we observed that 14 out of 15 variants with AG or DG DS > 0.20 resulted in predicted splicing effects, which allows us to conclude that the arbitrary threshold for AG or DG DS > 0.20 is very likely to predict the missplicing. However, further investigations that involve coding variants with AG or DG DSs < 0.20 are necessary to strengthen this conclusion.

In conclusion, we report novel missplicing events linked to missense and synonymous variants in *ABCA4* by implementing *in silico*-based splicing predictions by SpliceAI and *in vitro* splicing assays. The aberrant splicing allows a better understanding of the causality of the tested variants, and a more trustworthy severity assessment. The implications of these findings are significant, as they point toward novel treatment strategies that could be used for STGD1 individuals who carry these variants. Particularly, those involving synonymous variants that lead to splice aberrations may be amenable for splice modulating therapies. These results underscore the urgent need to further explore the pathomechanisms of other coding variants in *ABCA4* in order to better understand the manifestation of disease and develop and pursue new treatment approaches targeting STGD1.

## Materials and Methods

### SpliceAI for the selection of missense and synonymous variants for midigene splice assays

To select the missense and synonymous variants in *ABCA4* with a potential effect on splicing, the intronic regions were excluded from the analysis. Furthermore, variants affecting nucleotides located at the first, second, penultimate and ultimate position of each exon were not analyzed as these are most likely to interfere with splicing. The remaining coding nucleotides were filtered based on the assigned protein effect and only the missense or synonymous variants previously identified in STGD1 probands (therefore reported in LOVD; www.lovd.nl/ABCA4) that were previously (11) classified as ‘severe,’ ‘moderately severe’ or lacked the severity score were selected. These were assigned a delta score ranging from 0 (no predicted effect on splicing) to 1 (very likely effect on splicing) for acceptor gain (AG), acceptor loss (AL), donor gain (DG) and donor loss (DL) using SpliceAI. This study focused on those variants that were assigned a delta score > 0.20 for AG or DG. Variants c.4203C>A, c.4454C>T and c.4457C>T were assigned DS AG < 0.20, but were included in the analysis either as negative control or because of their localization near the cryptic SAS/SDS in exon 30 in *ABCA4*.

### Construction of *ABCA4* mutant midigenes

The variants c.1022A>T, c.3407G>T, c.3462C>T, c.3703A>G, c.4454C>T and c.4469G>A were introduced in appropriate wild-type BA clones (BA7, BA16, BA17, BA20) described previously (20) by using the NEBuilder HiFi DNA Assembly Master Mix (New England Biolabs, Ipswich, MA, USA) according to the manufacturer’s instructions. The WT plasmids served as template for amplification of two amplicons/WT plasmid with primers listed in Supplementary Material, Table S7. The PCR was performed by using the Phusion High-Fidelity DNA Polymerase (ThermoFisher Scientific, Waltham, MA, USA) by following the manufacturer’s instructions. Subsequently, the WT constructs were digested with two restriction enzymes, as follows: BA7 with *Pac*I (ThermoFisher Scientific, Waltham, MA, USA) and *Bsu*36I (ThermoFisher Scientific, Waltham, MA, USA), BA16 with *Bbv*CI (New England Biolabs, Ipswich, MA, USA) and *Bmg*BI (ThermoFisher Scientific, Waltham, MA, USA), BA17 with *Pfl*MI (ThermoFisher Scientific, Waltham, MA, USA) and *Sal*I (ThermoFisher Scientific, Waltham, MA, USA), and BA20 with *Bmg*BI and *Sal*I. The digested WT vector, the PCR amplicons and a synthetic dsDNA sequence (gBlock; Integrated DNA Technologies, Coralville, IA, USA) that incorporated one of the above-mentioned variants (Supplementary Material, Table S7) were introduced in the NEBuilder HiFi DNA Assembly Master Mix. The constructs were then used to transform NEB 10-beta competent cells (New England Biolabs, Ipswich, MA, USA).

To introduce the c.6207C>T and c.6272T>A variants in the WT BA28 construct, the latter was digested with *Blp*I (ThermoFisher Scientific, Waltham, MA, USA) and a 746-bp long dsDNA sequence (gBlock; Integrated DNA Technologies, Coralville, IA, USA) containing the *ABCA4* exon 45 with either one or the other variant and parts of the adjacent introns (1:94467791–1:94467046, GRCh37) was introduced as described previously (31). The constructs were used to transform GT115 competent cells (InvivoGen, San Diego, CA, USA).

The variants c.1977G>A, c.2128A>G, c.2273C>T, c.3096A>T, c.4203C>A, c.4446C>A, c.4457C>T, c.5088C>G, c.5367C>G and c.6339C>G were introduced in the appropriate WT BA constructs by mutagenesis using the primers reported in Table S8. For introducing the c.2128A>G and c.6339C>G variants, the forward and reverse primers were run separately in a PCR reaction with either BA12 or BA28 as template, respectively. Afterwards, 25 μL of the forward and reverse reactions were combined and set through the following cooling condition in order to allow reannealing of PCR products: 95°C for 5 min, 90°C for 1 min, 80°C for 1 min, 70°C for 30 s, 60°C for 30 s, 50°C for 30 s, 40°C for 30 s and final holding at 37°C. The mutated *ABCA4* sequences were placed in the pCI NEO (44) vector by the Gateway cloning system as described previously (38).

The mutant midigenes were linearized either with *Sal*I or *Age*I (New England Biolabs, Ipswich, MA, USA) and validated with PacBio sequencing.

### Splicing analysis

To identify the variants’ possible effect on splicing, HEK293T cells, once they reached 80% confluency, were transfected with both mutant or WT plasmid constructs at a concentration of 100 ng/well in 12-well plates. After 48 h, the cells were collected and their RNA was extracted. Subsequently, the extracted RNA was reverse-transcribed into cDNA using a previously reported method (38). To determine the isoform composition of the cDNA, RT-PCR was performed using the appropriate primer pairs reported in Supplementary Material, Table S9 following the previously reported protocol (20). The *ABCA4* transcripts were visualized using gel-electrophoresis on 2% agarose gels, and the bands were quantified using FIJI ImageJ 1.53c (details in Supplementary Material, Tables S4 and S5). The sequences of the detected *ABCA4* isoforms were confirmed by Sanger sequencing.

### Updating the ACMG/AMP classification of analyzed variants

Updating the ACMG/AMP classification of the variants reported in this study was conducted by referencing the previous comprehensive categorization of *ABCA4* variants carried out by Cornelis and colleagues (22). Specifically, Cornelis *et al*. labeled the severity of all *ABCA4* variants present in 5579 biallelic STGD1 probands by following the guidelines suggested by Richards *et al*. (19) and coupling them with the point system developed by Tavtigian *et al*. (45).

The variants that resulted in aberrant splicing were labeled with the PS3_Moderate label according to the ACMG/AMP guidelines. Table 3 reports instances where the addition of this label led to a higher ACMG/AMP class based on the point system.

## Supplementary Material

Supplementary_data_ddad129

Table_S1_ddad129

Table_S2_ddad129

Table_S3_ddad129

Table_S4_ddad129

Table_S5_ddad129

Table_S6_ddad129

## Data Availability

Raw data generated for this paper are available upon request from corresponding author.

## References

[1] Allikmets, R., Singh, N., Sun, H., Shroyer, N.F., Hutchinson, A., Chidambaram, A., Gerrard, B., Baird, L., Stauffer, D., Peiffer, A. et al. (1997) A photoreceptor cell-specific ATP-bindingtransporter gene (ABCR) is mutated in recessive Starqardt macular dystrophy. Nat. Genet., 15, 236–246. 9054934 10.1038/ng0397-236

[2] Quazi, F., Lenevich, S. and Molday, R.S. (2012) ABCA4 is an N-retinylidene-phosphatidylethanolamine and phosphatidylethanolamine importer. Nat. Commun., 3, 925. 22735453 10.1038/ncomms1927 PMC3871175

[3] Burke, T.R., Duncker, T., Woods, R.L., Greenberg, J.P., Zernant, J., Tsang, S.H., Smith, R.T., Allikmets, R., Sparrow, J.R. and Delori, F.C. (2014) Quantitative fundus autofluorescence in recessive Stargardt disease. Invest. Ophthalmol. Vis. Sci., 55, 2841–2852. 24677105 10.1167/iovs.13-13624 PMC4008047

[4] Sparrow, J.R. and Boulton, M. (2005) RPE lipofuscin and its role in retinal pathobiology. Exp. Eye Res., 80, 595–606. 15862166 10.1016/j.exer.2005.01.007

[5] Sparrow, J.R., Wu, Y., Kim, C.Y. and Zhou, J. (2010) Phospholipid meets all-trans-retinal: the making of RPE bisretinoids. J. Lipid Res., 51, 247–261. 19666736 10.1194/jlr.R000687 PMC2803227

[6] Sparrow, J.R. and Yamamoto, K. (2012) The bisretinoids of RPE lipofuscin: a complex mixture. Adv. Exp. Med. Biol., 723, 761–767. 22183404 10.1007/978-1-4614-0631-0_97 PMC11829280

[7] Cremers, F.P.M., Lee, W., Collin, R.W.J. and Allikmets, R. (2020) Clinical spectrum, genetic complexity and therapeutic approaches for retinal disease caused by ABCA4 mutations. Prog. Retin. Eye Res., 79, 100861. 32278709 10.1016/j.preteyeres.2020.100861 PMC7544654

[8] Runhart, E.H., Khan, M., Cornelis, S.S., Roosing, S., Del Pozo-Valero, M., Lamey, T.M., Liskova, P., Roberts, L., Stohr, H., Klaver, C.C.W. et al. (2020) Association of sex with frequent and mild ABCA4 alleles in Stargardt disease. JAMA Ophthalmol., 138, 1035–1042. 32815999 10.1001/jamaophthalmol.2020.2990 PMC7441467

[9] Runhart, E.H., Sangermano, R., Cornelis, S.S., Verheij, J., Plomp, A.S., Boon, C.J.F., Lugtenberg, D., Roosing, S., Bax, N.M., Blokland, E.A.W. et al. (2018) The common ABCA4 variant p.Asn1868Ile shows nonpenetrance and variable expression of Stargardt disease when present in trans with severe variants. Invest. Ophthalmol. Vis. Sci., 59, 3220–3231. 29971439 10.1167/iovs.18-23881

[10] Zernant, J., Lee, W., Wang, J., Goetz, K., Ullah, E., Nagasaki, T., Su, P.Y., Fishman, G.A., Tsang, S.H., Tumminia, S.J. et al. (2022) Rare and common variants in ROM1 and PRPH2 genes trans-modify Stargardt/ABCA4 disease. PLoS Genet., 18, e1010129. 35353811 10.1371/journal.pgen.1010129 PMC9000055

[11] Cornelis, S.S., Runhart, E.H., Bauwens, M., Corradi, Z., De Baere, E., Roosing, S., Haer-Wigman, L., Dhaenens, C.M., Vulto-van Silfhout, A.T. and Cremers, F.P. (2022) Personalized genetic counseling for Stargardt disease: offspring risk estimates based on variant severity. Am. J. Hum. Genet., 109, 498–507. 35120629 10.1016/j.ajhg.2022.01.008 PMC8948157

[12] Curtis, S.B., Molday, L.L., Garces, F.A. and Molday, R.S. (2020) Functional analysis and classification of homozygous and hypomorphic ABCA4 variants associated with Stargardt macular degeneration. Hum. Mutat., 41, 1944–1956. 32845050 10.1002/humu.24100 PMC7755071

[13] Garces, F., Jiang, K., Molday, L.L., Stohr, H., Weber, B.H., Lyons, C.J., Maberley, D. and Molday, R.S. (2018) Correlating the expression and functional activity of ABCA4 disease variants with the phenotype of patients with Stargardt disease. Invest. Ophthalmol. Vis. Sci., 59, 2305–2315. 29847635 10.1167/iovs.17-23364 PMC5937799

[14] Garces, F.A., Scortecci, J.F. and Molday, R.S. (2020) Functional characterization of ABCA4 missense variants linked to Stargardt macular degeneration. Int. J. Mol. Sci., 22, 185. 33375396 10.3390/ijms22010185 PMC7796138

[15] Molday, R.S., Garces, F.A., Scortecci, J.F. and Molday, L.L. (2021) Structure and function of ABCA4 and its role in the visual cycle and Stargardt macular degeneration. Prog. Retin. Eye. Res., 89, 101036. 34954332 10.1016/j.preteyeres.2021.101036

[16] Sun, H., Smallwood, P.M. and Nathans, J. (2000) Biochemical defects in ABCR protein variants associated with human retinopathies. Nat. Genet., 26, 242–246. 11017087 10.1038/79994

[17] Jaganathan, K., Kyriazopoulou Panagiotopoulou, S., McRae, J.F., Darbandi, S.F., Knowles, D., Li, Y.I., Kosmicki, J.A., Arbelaez, J., Cui, W., Schwartz, G.B. et al. (2019) Predicting splicing from primary sequence with deep learning. Cell, 176, 535–548.e24. 30661751 10.1016/j.cell.2018.12.015

[18] Riepe, T.V., Khan, M., Roosing, S., Cremers, F.P.M. and t’hoen, P.A.C. (2021) Benchmarking deep learning splice prediction tools using functional splice assays. Hum. Mutat., 42, 799–810. 33942434 10.1002/humu.24212 PMC8360004

[19] Richards, S., Aziz, N., Bale, S., Bick, D., Das, S., Gastier-Foster, J., Grody, W.W., Hegde, M., Lyon, E., Spector, E. et al. (2015) Standards and guidelines for the interpretation of sequence variants: a joint consensus recommendation of the American College of Medical Genetics and Genomics and the Association for Molecular Pathology. Genet. Med., 17, 405–424. 25741868 10.1038/gim.2015.30 PMC4544753

[20] Sangermano, R., Khan, M., Cornelis, S.S., Richelle, V., Albert, S., Garanto, A., Elmelik, D., Qamar, R., Lugtenberg, D., van den Born, L.I., Collin, R.W.J. and Cremers, F.P.M. (2018) ABCA4 midigenes reveal the full splice spectrum of all reported noncanonical splice site variants in Stargardt disease. Genome Res., 28, 100–110. 29162642 10.1101/gr.226621.117 PMC5749174

[21] Huang, D., Thompson, J.A., Chen, S.C., Adams, A., Pitout, I., Lima, A., Zhang, D., Jeffery, R.C.H., Attia, M.S., McLaren, T.L. et al. (2022) Characterising splicing defects of ABCA4 variants within exons 13-50 in patient-derived fibroblasts. Exp. Eye Res., 225, 109276. 36209838 10.1016/j.exer.2022.109276

[22] Cornelis, S.S., Bauwens, M., Haer-Wigman, L., Bruyne, M.D., Pantrangi, M., Baere, E.D., Hufnagel, R.B., Dhaenens, C.-M. and Cremers, F.P.M. (2023) Compendium of clinical variant classification for 2,247 unique ABCA4 variants to improve genetic medicine access for Stargardt disease. medRxiv, 2023.04.24.23288782. 10.1155/2023/6815504 PMC11918811 40225145

[23] Bass, B.L. and Weintraub, H. (1987) A developmentally regulated activity that unwinds RNA duplexes. Cell, 48, 607–613. 2434241 10.1016/0092-8674(87)90239-x

[24] Bass, B.L. and Weintraub, H. (1988) An unwinding activity that covalently modifies its double-stranded RNA substrate. Cell, 55, 1089–1098. 3203381 10.1016/0092-8674(88)90253-x

[25] Rebagliati, M.R. and Melton, D.A. (1987) Antisense RNA injections in fertilized frog eggs reveal an RNA duplex unwinding activity. Cell, 48, 599–605. 2434240 10.1016/0092-8674(87)90238-8

[26] Schneider, N., Sundaresan, Y., Gopalakrishnan, P., Beryozkin, A., Hanany, M., Levanon, E.Y., Banin, E., Ben-Aroya, S. and Sharon, D. (2022) Inherited retinal diseases: linking genes, disease-causing variants, and relevant therapeutic modalities. Prog. Retin. Eye Res., 89, 101029. 34839010 10.1016/j.preteyeres.2021.101029

[27] Sheridan, C. (2023) Shoot the messenger: RNA editing is here. Nat. Biotechnol., 41, 306–308. 10.1038/s41587-023-01709-8 36879010

[28] De Angeli, P., Reuter, P., Hauser, S., Schols, L., Stingl, K., Wissinger, B. and Kohl, S. (2022) Effective splicing restoration of a deep-intronic ABCA4 variant in cone photoreceptor precursor cells by CRISPR/SpCas9 approaches. Mol. Ther. Nucleic Acids, 29, 511–524. 35991315 10.1016/j.omtn.2022.07.023 PMC9375153

[29] Albert, S., Garanto, A., Sangermano, R., Khan, M., Bax, N.M., Hoyng, C.B., Zernant, J., Lee, W., Allikmets, R., Collin, R.W.J. and Cremers, F.P.M. (2018) Identification and rescue of splice defects caused by two neighboring deep-intronic ABCA4 mutations underlying Stargardt disease. Am. J. Hum. Genet., 102, 517–527. 29526278 10.1016/j.ajhg.2018.02.008 PMC5985352

[30] Garanto, A., Duijkers, L., Tomkiewicz, T.Z. and Collin, R.W.J. (2019) Antisense oligonucleotide screening to optimize the rescue of the splicing defect caused by the recurrent deep-intronic ABCA4 variant c.4539+2001G>a in Stargardt disease. Genes (Basel), 10, 452. 31197102 10.3390/genes10060452 PMC6628380

[31] Kaltak, M., de Bruijn, P., Piccolo, D., Lee, S.-E., Dulla, K., Hoogenboezem, T., Beumer, W., Webster, A.R., Collin, R.W.J., Cheetham, M.E., Platenburg, G. and Swildens, J. (2023) Antisense oligonucleotide therapy corrects splicing in the common Stargardt disease type 1-causing variant ABCA4 c.5461-10T>C. Mol. Ther. Nucleic Acids, 31, 674–688. 36910710 10.1016/j.omtn.2023.02.020 PMC9999166

[32] Khan, M., Arno, G., Fakin, A., Parfitt, D.A., Dhooge, P.P.A., Albert, S., Bax, N.M., Duijkers, L., Niblock, M., Hau, K.L. et al. (2020) Detailed phenotyping and therapeutic strategies for intronic ABCA4 variants in Stargardt disease. Mol. Ther. Nucleic Acids, 21, 412–427. 32653833 10.1016/j.omtn.2020.06.007 PMC7352060

[33] Sangermano, R., Garanto, A., Khan, M., Runhart, E.H., Bauwens, M., Bax, N.M., van den Born, L.I., Khan, M.I., Cornelis, S.S., Verheij, J. et al. (2019) Deep-intronic ABCA4 variants explain missing heritability in Stargardt disease and allow correction of splice defects by antisense oligonucleotides. Genet. Med., 21, 1751–1760. 30643219 10.1038/s41436-018-0414-9 PMC6752325

[34] Tomkiewicz, T.Z., Nieuwenhuis, S.E., Cremers, F.P.M., Garanto, A. and Collin, R.W.J. (2022) Correction of the splicing defect caused by a recurrent variant in ABCA4 (c.769-784C>T) that underlies Stargardt disease. Cell, 11, 3947. 10.3390/cells11243947 PMC9777113 36552712

[35] Tomkiewicz, T.Z., Suarez-Herrera, N., Cremers, F.P.M., Collin, R.W.J. and Garanto, A. (2021) Antisense oligonucleotide-based rescue of aberrant splicing defects caused by 15 pathogenic variants in ABCA4. Int. J. Mol. Sci., 22, 4621. 33924840 10.3390/ijms22094621 PMC8124656

[36] Sabirzhanova, I., Lopes Pacheco, M., Rapino, D., Grover, R., Handa, J.T., Guggino, W.B. and Cebotaru, L. (2015) Rescuing trafficking mutants of the ATP-binding cassette protein, ABCA4, with small molecule correctors as a treatment for Stargardt eye disease. J. Biol. Chem., 290, 19743–19755. 26092729 10.1074/jbc.M115.647685 PMC4528136

[37] Ramalho, A.S., Boon, M., Proesmans, M., Vermeulen, F., Carlon, M.S. and Boeck, K. (2022) Assays of CFTR function in vitro, ex vivo and in vivo. Int. J. Mol. Sci., 23, 1437. 35163362 10.3390/ijms23031437 PMC8836180

[38] Sangermano, R., Bax, N.M., Bauwens, M., van den Born, L.I., De Baere, E., Garanto, A., Collin, R.W., Goercharn-Ramlal, A.S., den Engelsman-van Dijk, A.H., Rohrschneider, K. et al. (2016) Photoreceptor progenitor mRNA analysis reveals exon skipping resulting from the ABCA4 c.5461-10T-->C mutation in Stargardt disease. Ophthalmology, 123, 1375–1385. 26976702 10.1016/j.ophtha.2016.01.053

[39] Vazquez-Dominguez, I., Duijkers, L., Fadaie, Z., Alaerds, E.C.W., Post, M.A., van Oosten, E.M., O'Gorman, L., Kwint, M., Koolen, L., Hoogendoorn, A.D.M. et al. (2022) The predicted splicing variant c.11+5G>a in RPE65 leads to a reduction in mRNA expression in a cell-specific manner. Cell, 11, 3640. 10.3390/cells11223640 PMC9688607 36429068

[40] Vazquez-Dominguez, I., Li, C.H.Z., Fadaie, Z., Haer-Wigman, L., Cremers, F.P.M., Garanto, A., Hoyng, C.B. and Roosing, S. (2022) Identification of a complex allele in IMPG2 as a cause of adult-onset vitelliform macular dystrophy. Invest. Ophthalmol. Vis. Sci., 63, 27. 10.1167/iovs.63.5.27 PMC9150824 35608844

[41] Zhang, C., Hastings, M.L., Krainer, A.R. and Zhang, M.Q. (2007) Dual-specificity splice sites function alternatively as 5′ and 3′ splice sites. Proc. Natl. Acad. Sci. U. S. A., 104, 15028–15033. 17848517 10.1073/pnas.0703773104 PMC1986607

[42] Moles-Fernandez, A., Domenech-Vivo, J., Tenes, A., Balmana, J., Diez, O. and Gutierrez-Enriquez, S. (2021) Role of splicing regulatory elements and in silico tools usage in the identification of deep intronic splicing variants in hereditary breast/ovarian cancer genes. Cancers (Basel), 13, 3341. 34283047 10.3390/cancers13133341 PMC8268271

[43] Collin, R.W., de Heer, A.M., Oostrik, J., Pauw, R.J., Plantinga, R.F., Huygen, P.L., Admiraal, R., de Brouwer, A.P., Strom, T.M., Cremers, C.W. and Kremer, H. (2008) Mid-frequency DFNA8/12 hearing loss caused by a synonymous TECTA mutation that affects an exonic splice enhancer. Eur. J. Hum. Genet., 16, 1430–1436. 18575463 10.1038/ejhg.2008.110

[44] Gamundi, M.J., Hernan, I., Muntanyola, M., Maseras, M., Lopez-Romero, P., Alvarez, R., Dopazo, A., Borrego, S. and Carballo, M. (2008) Transcriptional expression of cis-acting and trans-acting splicing mutations cause autosomal dominant retinitis pigmentosa. Hum. Mutat., 29, 869–878. 18412284 10.1002/humu.20747

[45] Tavtigian, S.V., Greenblatt, M.S., Harrison, S.M., Nussbaum, R.L., Prabhu, S.A., Boucher, K.M., Biesecker, L.G. and ClinGen Sequence Variant Interpretation Working Group (2018) Modeling the ACMG/AMP variant classification guidelines as a Bayesian classification framework. Genet. Med., 20, 1054–1060. 29300386 10.1038/gim.2017.210 PMC6336098

