# Supplementary material for: Stargardt disease-associated missense and synonymous ABCA4 variants result in aberrant splicing
Source: Hum Mol Genet. 2023 Aug 9;32(21):3078–89. doi: 10.1093/hmg/ddad129 (PMC10586196; doi:10.1093/hmg/ddad129)
Supplement: Supplementary_data_ddad129 [file supplementary_data_ddad129.pdf]

SUPPLEMENTARY DATA

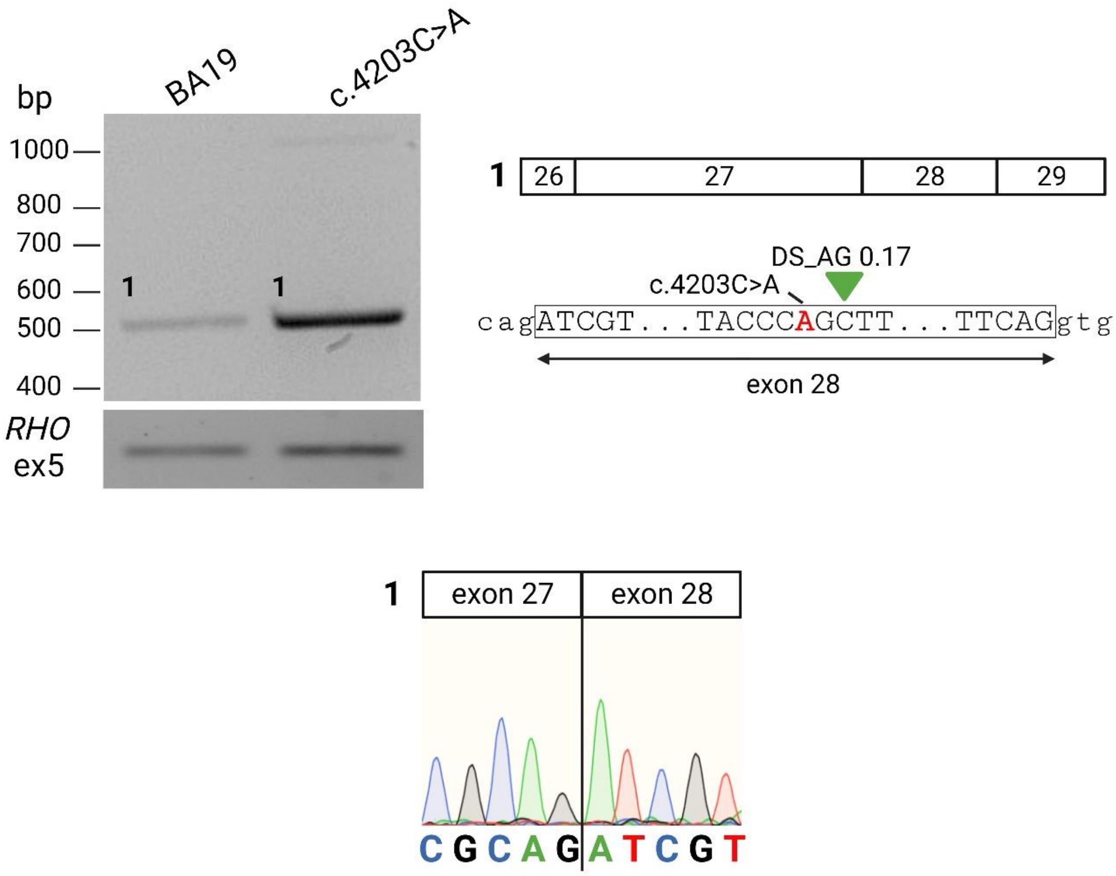

Figure S1. RT-PCR of the c.4203C>A sample. The BA19 construct served as control. The identified *ABCA4* isoforms were validated by Sanger sequencing. No deviant splicing events were observed when compared to the BA19 WT plasmid.

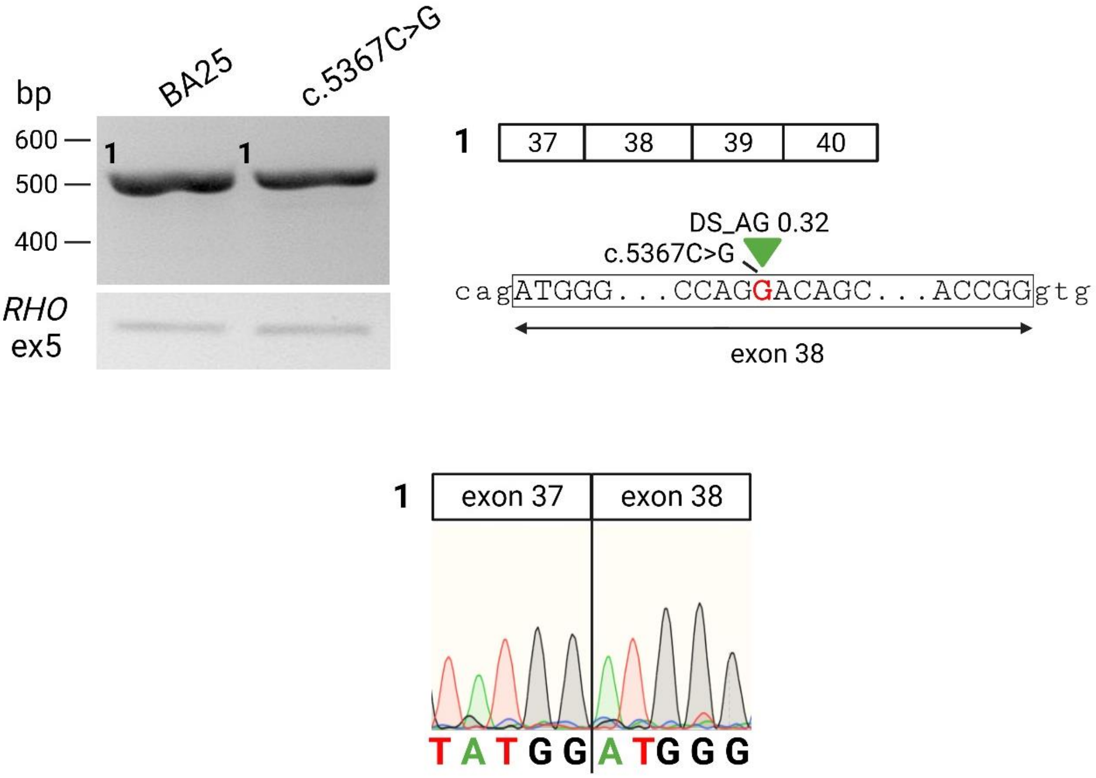

Figure S2. RT-PCR of the c.5367C>G sample. The BA25 construct served as control. The RNA of mutant midigene-transfected HEK293T cells express the correctly spliced isoform, as in BA25 WT. The sequences of identified *ABCA4* transcripts were validated by Sanger sequencing.

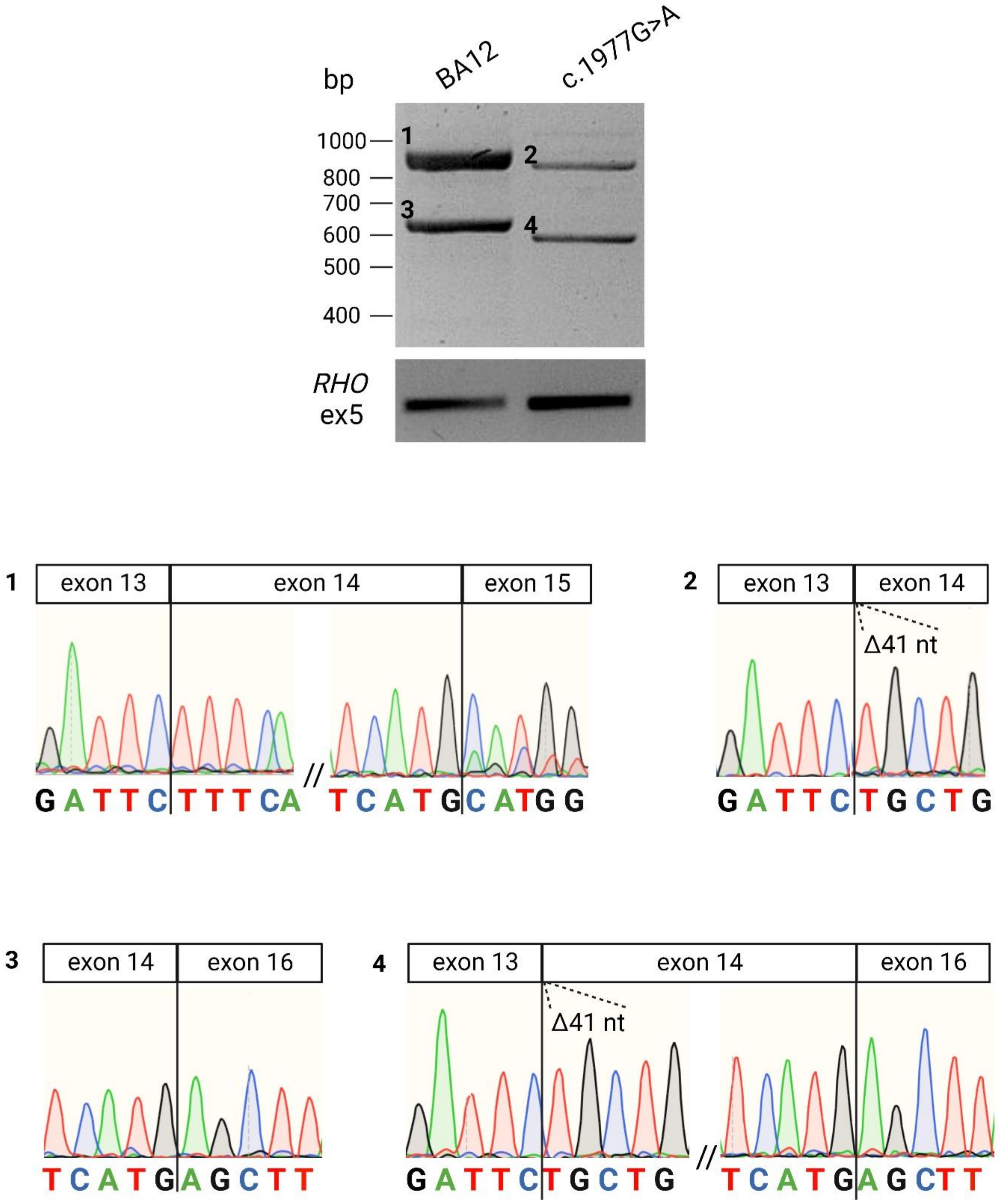

Figure S3. RT-PCR of the c.1977G>A sample upon plasmid transfection in HEK293T cells. The BA12 construct served as control. The sequences of detected *ABCA4* isoforms were confirmed by Sanger sequencing.

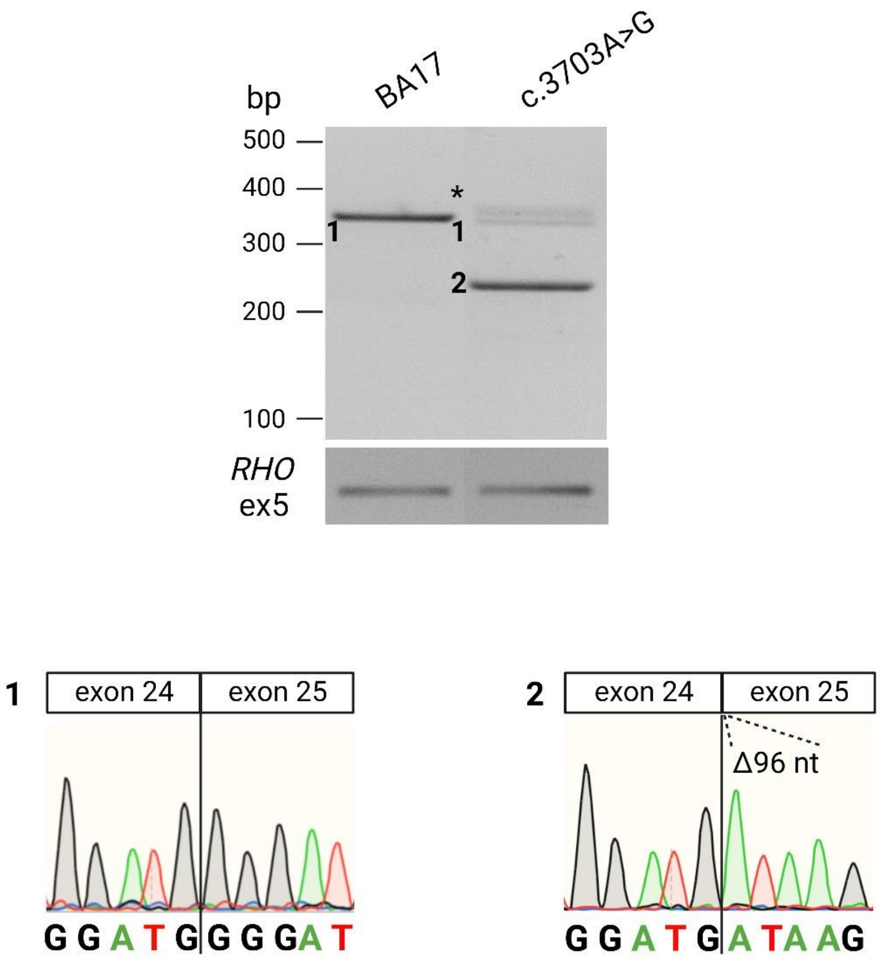

Figure S4. RT-PCR of the c.3703A>G sample upon plasmid transfection in HEK293T cells. The BA17 construct served as control. The sequences of detected *ABCA4* isoforms were confirmed by Sanger sequencing.

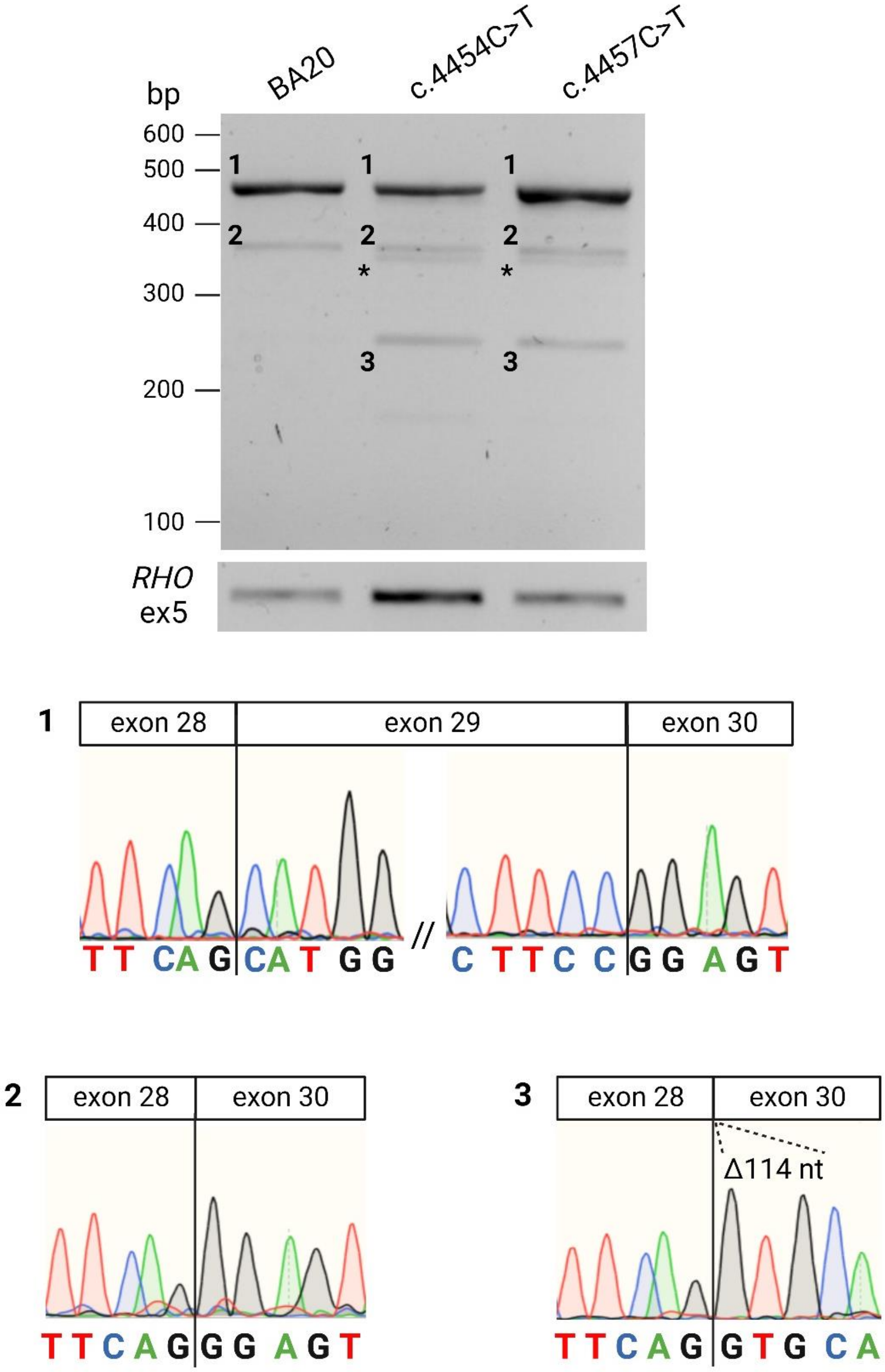

Figure S5. RT-PCR of the c.4454C>T and c.4454C>T samples upon plasmid transfection in HEK293T cells. The BA20 construct served as negative control. The sequences of detected *ABCA4* isoforms were confirmed by Sanger sequencing.

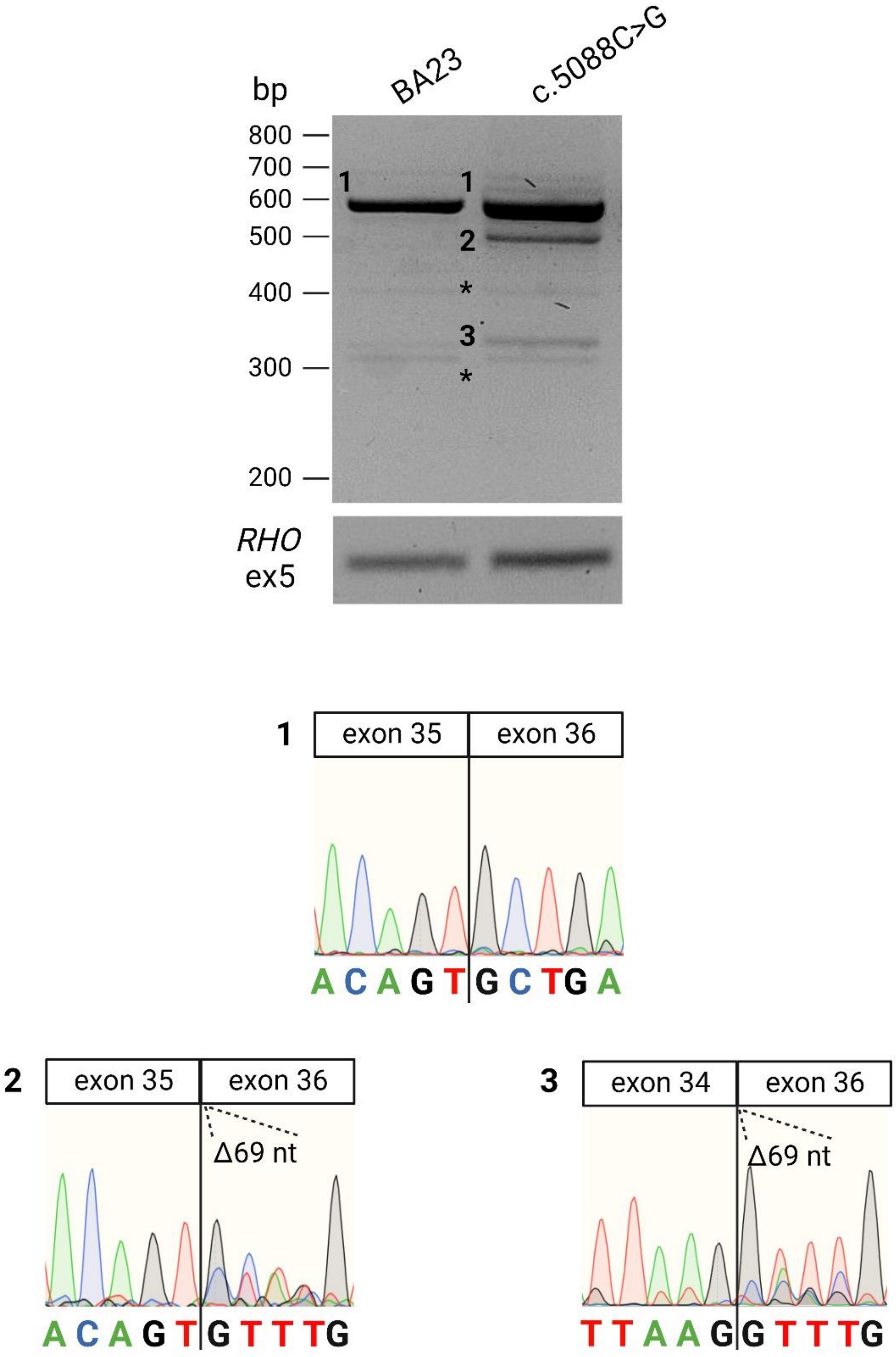

Figure S6. RT-PCR of the c.5088C>G sample upon plasmid transfection in HEK293T cells. The BA23 construct served as negative control. The sequences of detected *ABCA4* isoforms were confirmed by Sanger sequencing.

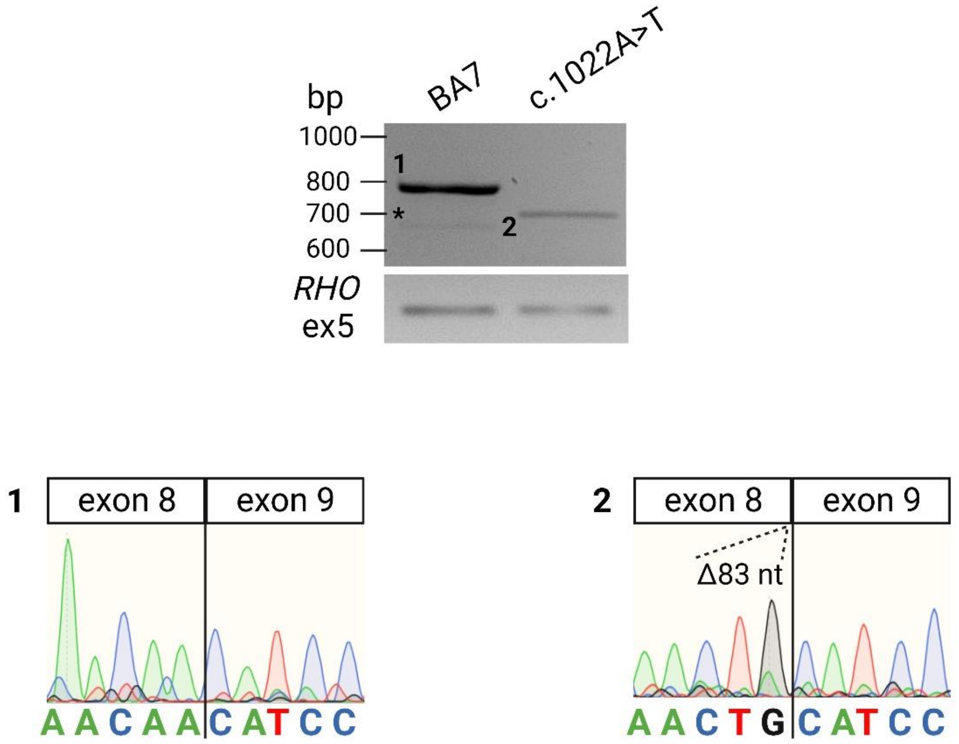

Figure S7. RT-PCR of the c.1022A>T sample upon plasmid transfection in HEK293T cells. The BA7 construct served as control. The sequences of detected *ABCA4* isoforms were confirmed by Sanger sequencing.

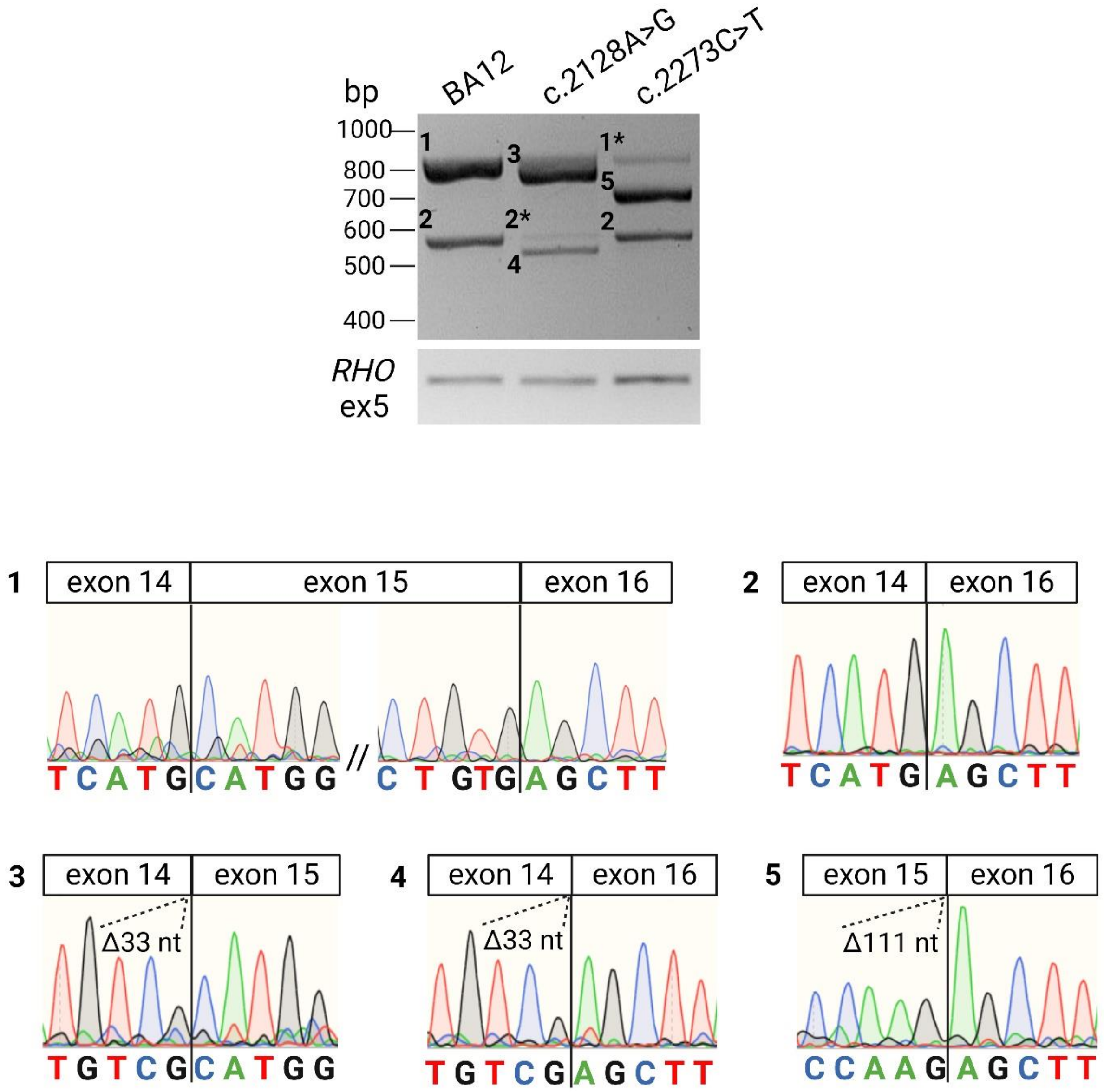

Figure S8. RT-PCR of the c.2128A>G and c.2273C>T samples upon plasmid transfection in HEK293T cells. The BA12 construct served as control. The sequences of detected *ABCA4* isoforms were confirmed by Sanger sequencing.

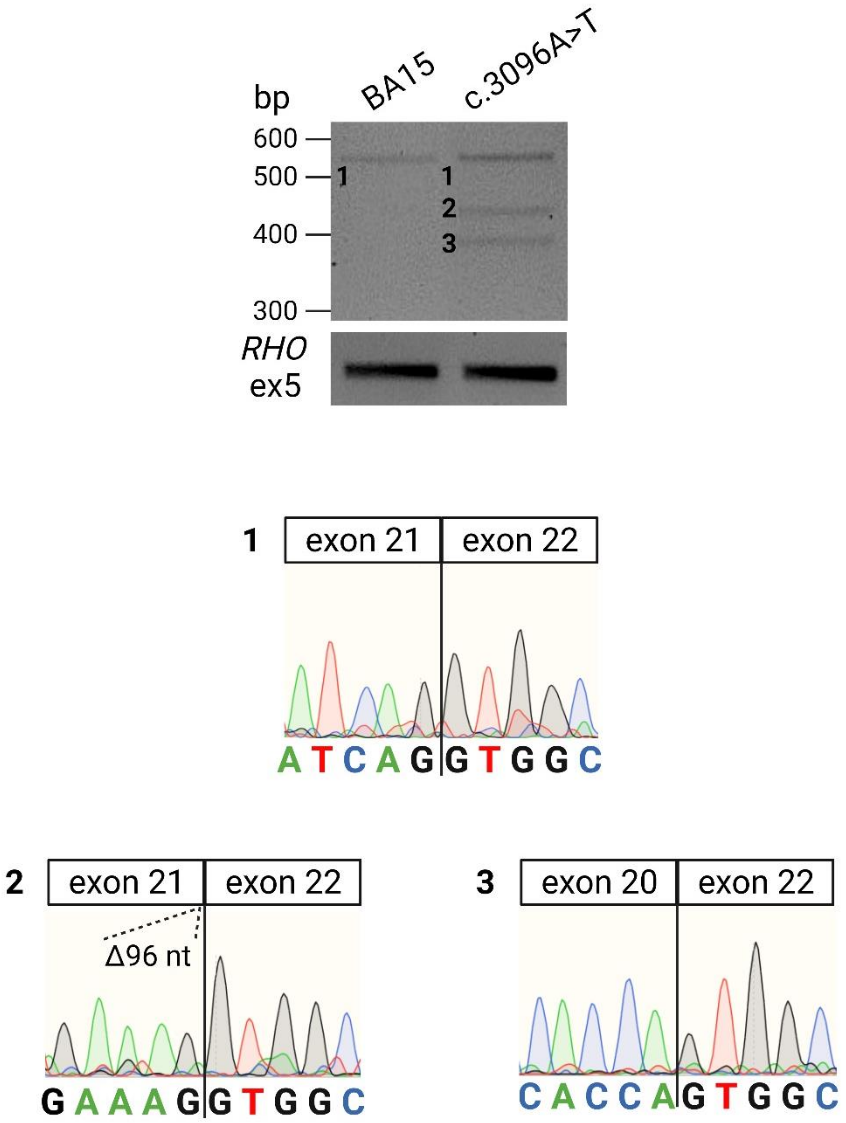

Figure S9. RT-PCR of the c.3096A>T sample upon plasmid transfection in HEK293T cells. The BA15 construct served as control. The sequences of detected *ABCA4* isoforms were confirmed by Sanger sequencing.

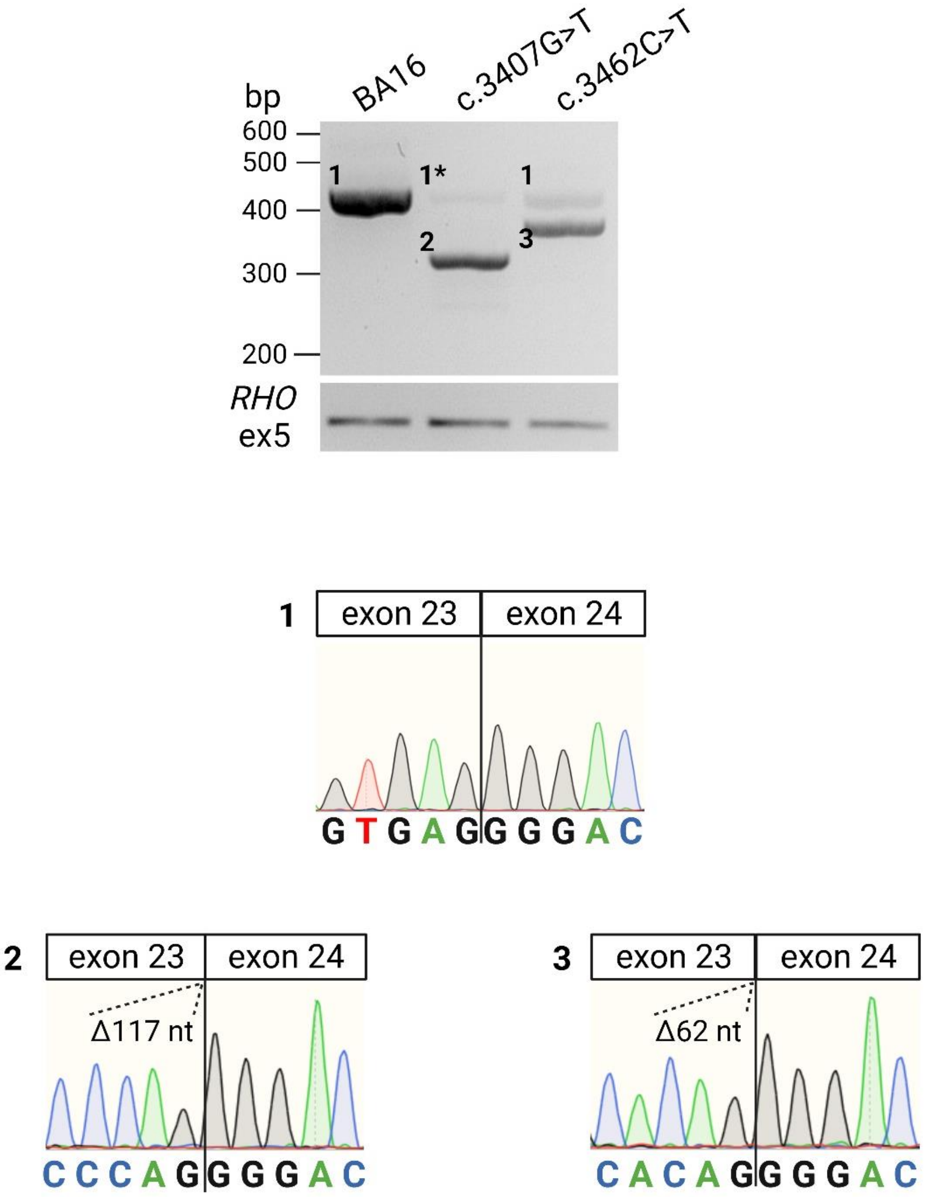

Figure S10. RT-PCR of the c.3407G>T and c.3462C>T samples upon plasmid transfection in HEK293T cells. The BA16 construct served as control. The sequences of detected *ABCA4* isoforms were confirmed by Sanger sequencing.

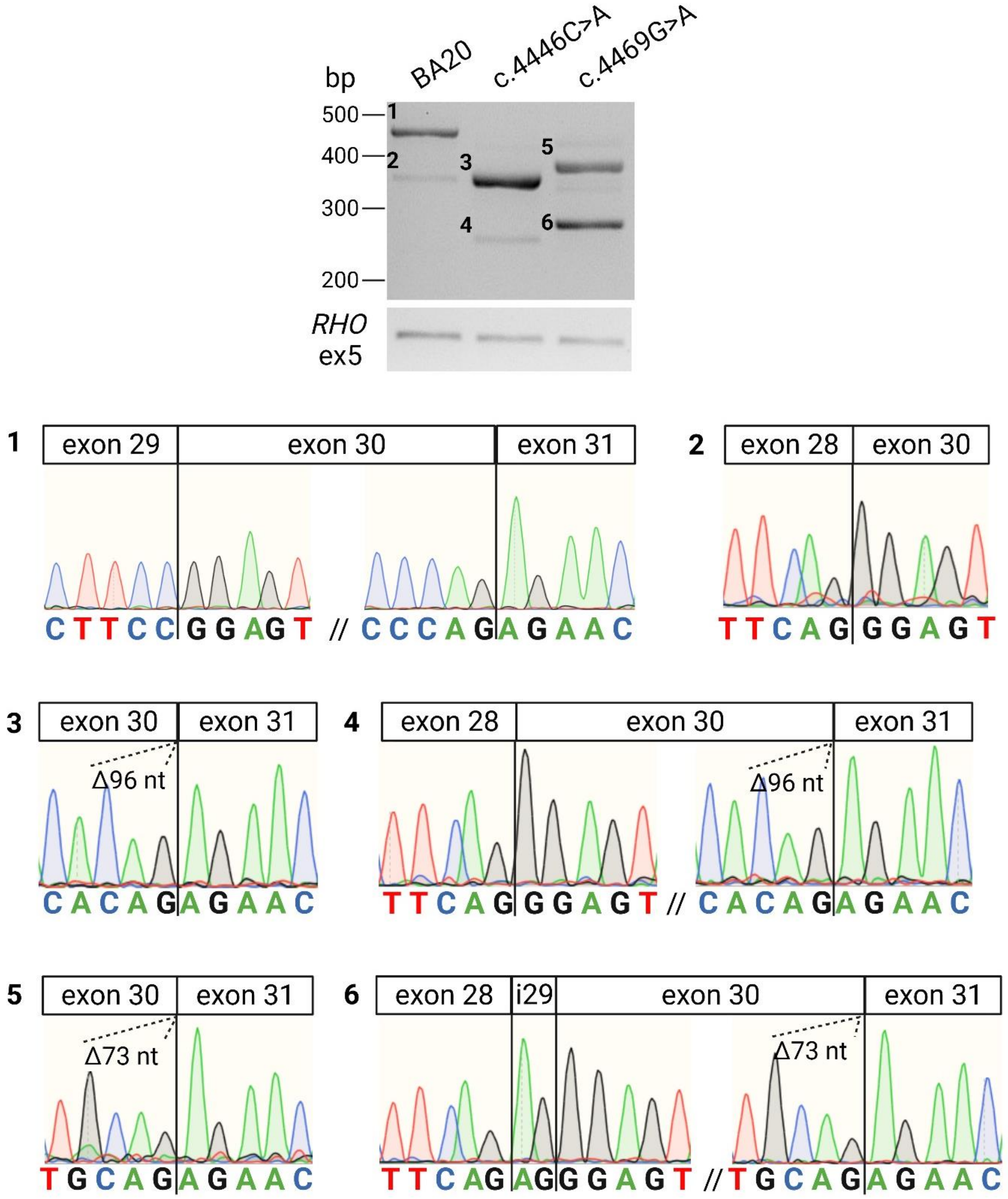

Figure S11. RT-PCR of the c.4446C>A and c.4469G>A samples upon plasmid transfection in HEK293T cells. The BA20 construct served as control. The sequences of detected *ABCA4* isoforms were confirmed by Sanger sequencing.

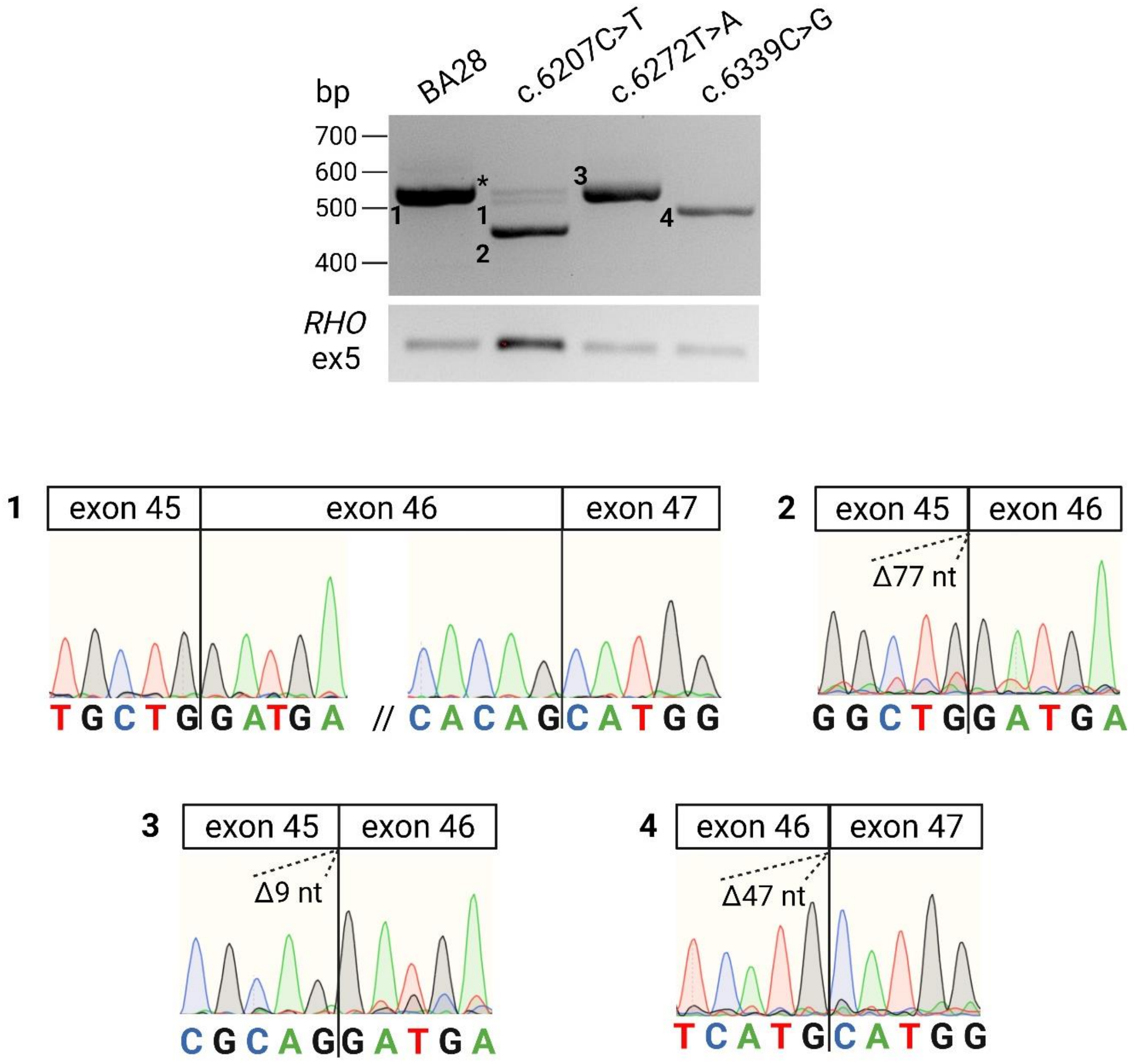

Figure S12. RT-PCR of the c.6207C>T, c.6272T>A and c.6339C>G samples upon plasmid transfection in HEK293T cells. The BA28 construct served as control. The sequences of detected *ABCA4* isoforms were confirmed by Sanger sequencing.

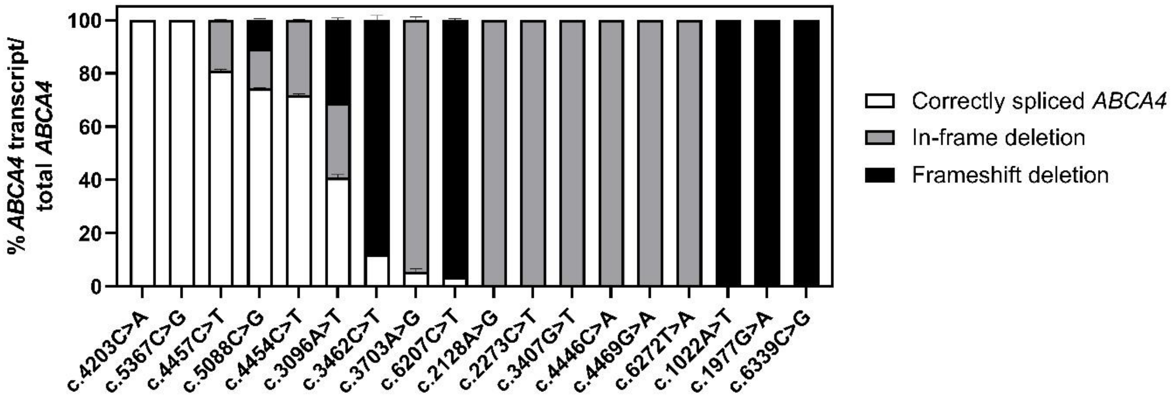

Figure S13. Overview of all detected splicing events upon RT-PCR of midigene-transfected HEK293T cells. Data is shown as mean±SEM.

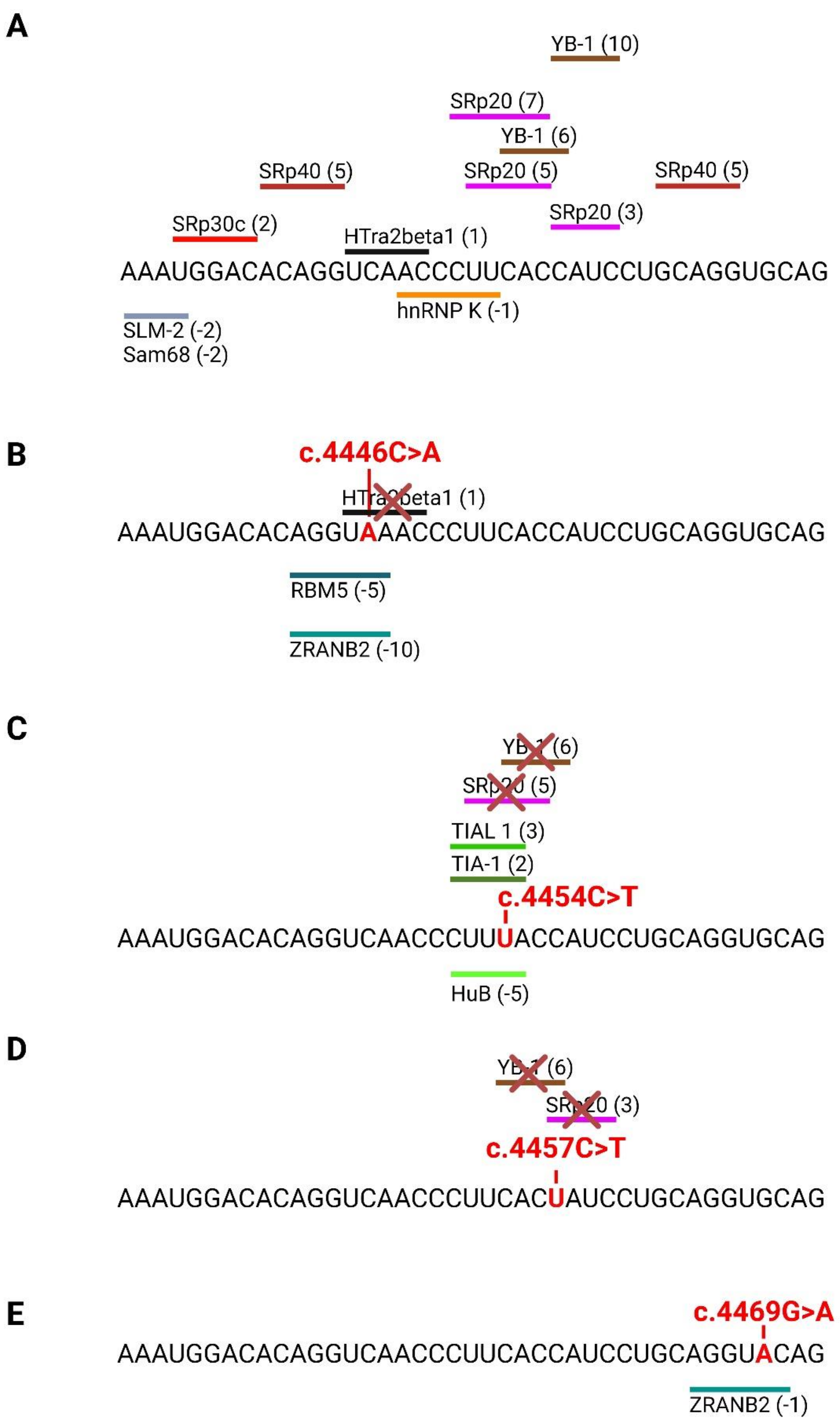

Figure S14. Overview of exonic splicing regulatory sequences in part of *ABCA4* exon 30 (94 945 108 – 94 945 067, GRCh37). (A) The exonic splicing regulatory (ESR) sequences in wild-type *ABCA4* mRNA exon 30 alter in the presence of (B) c.4446C>A, (C) c.4454C>T, (D) c.4457C>T and (E) c.4469G>A variants. The exonic splice enhancers (ESEs) are depicted

with scores 1-10, while the exonic splice silencers (ESSs) are represented with values between -1 and -10 (http://www.introni.it/, accessed 31 May 2023).

Table S7. List of primers (uppercase characters) and gBlocks (lowercase characters) used to introduce the variants in wild-type BA plasmids with HiFi DNA Assembly Mix.

WT construct	Variant	Sequence (5' – 3')	Length amplicon or gBlock (bp)
**BA7**	c.1022A>T	AAAGATGCGCACATTTTAATTAACATCTGATTGCACAAGTCTCCTCCC (FW)	2179
		ATACTCGGCCGATGGATAAACTAGGGCAAGGCAAAGTCTTCAG (RV)	
		CCTATCTATTCTTATGACAGAAGAACAAGTAAGTTTTCTGAGTCCTGCTTATAAATTGGC (FW)	168
		CTGGGAGAAGTCTCTTTACCTAAGGCCACTCAGCAAGACAAGAC (RV)	
		tttatccatcggccgagtatgcaggacttgctgtgggtgaccaggcccctcatgcagaatggtggtccagagacctttacaaagctgatgggcatcctgtctgacctcctgtgtggctaccccgagggaggtggctctcgggtgctctccttcaactggtatgtagacaataactataaggcctttctggggattgactccacaaggaaggatcctatctattcttatgacagaagaacaa	241
**BA16**	c.3407G>T ; c.3462C>T	GTTCGAGCCATTCTCCTGCCTCAGCCTCCTGAGTAGCTGGGACT (FW)	641
		GTGGACATGATGATGGTTCTGCCTGCAAGGTAGGGGCCAGG (RV)	
		GCCAAAGGAAAGGCAGTGAGGTAGGTGTCTGCCCAGG (FW)	1172
		TCTGGAGTTAGGTCATCGACGTGGGCTGGACACGTGGTGG (RV)	
	c.3407G>T	gcagaaccatcatcatgtccactcaccacatggacgaggccgacctccttggggaccgcattgccatcattgcccaggtaaggctctactgctcaggcaccccactcttcctgaagaactgctttggcacaggcttgtacttaaccttggtgcgcaagatgaaaaacatccagagccaaaggaaaggcagtgag	194
	c.3462C>T	Gcagaaccatcatcatgtccactcaccacatggacgaggccgacctccttggggaccgcattgccatcattgcccagggaaggctctactgctcaggcaccccactcttcctgaagaactgctttggcacaggtttgtacttaaccttggtgcgcaagatgaaaaacatccagagccaaaggaaaggcagtgag	194
**BA17**	c.3703A>G	ACCTCACAGTCTTCCAGTTTCTGGAAAACAGAGCTGGCATCAGT (FW)	121
		CCACCAGCTTTGCCTCTGGAACATGGTGGAGAACTACATCCAT (RV)	
		GACACTCCCCTGGAAGAGGTAAAGTAGAGATTCCAGCTGGTTTCTGTCA (FW)	2136

		CTAAAGGGAAGCGGCCGCCCGGGTCGACAGGTGTAGG GGATGGGAG (RV)	
		agaggcaaagctggtggagtgcattggtcaagaacttatcttccttcttccagataa gaacttcaagcacagagcatatgccagccttttcagagagctggaggagacgctg gctgaccttggtctcagcagttttggaatttctgacactcccctggaagag	163
**BA20**	c.4454C>T ; c.4469G>A	GCCACAGTCATGTTTATTCCACGTCTATTCTCCCACAGA TCGTGCTCCC (FW)	1549
		TGAGTTGCCACAGGGGTACTCCCTCATGGAAGACAAGA AAATATTCCATAATCAGCCT (RV)	
		AGCACCAGGGAGAAGCTCACCATGCTGCCAGAGTGCCC C (FW)	1037
		GCACATGGAGAGCTTCTTAATTAAGACTGTTAATAAGTG TGTAAACACATTTCC (RV)	
	c.4454C>T	ggagtacccctgtggcaactcaacaccctggaagactccttctgtgtccccaaaca tcacccagctgttccagaagcagaaatggacacaggtcaacccctttaccatcctgc aggtgcagcaccagggagaagctcacc	139
	c.4469G>A	ggagtacccctgtggcaactcaacaccctggaagactccttctgtgtccccaaaca tcacccagctgttccagaagcagaaatggacacaggtcaacccctttcaccatcctg caggtacagcaccagggagaagctcacc	139

Table S8. List of primers used for introducing the missense variants in wild-type midigenes by mutagenesis.

WT construct	Variant	Sequence (5' – 3')
BA12	c.1977G>A	ACCGCTGTTTCCCTATCTTCATAGTGCTGGCATGG (FW)
		CCATGCCAGCACTATGAAGATAGGGAAACAGCGGT (RV)
	c.2128A>G	GCTTCTCCATCATGTCGGTGAGCATCTTCCTCCTG (FW)
		CAGGAGGAAGATGCTCACCGACATGATGGAGAAGC (RV)
	c.2273C>T	CCTTCTTCTCCAAGGTCAGTCTGGCAGCAGC (FW)
		GCTGCTGCCAGACTGACCTTGGAGAAGAAGG (RV)
BA19	c.4203C>A	CTTTTGGCGAATACCCAGCTTTGACCCTTCACC (FW)
		GGTGAAGGGTCAAAGCTGGGTATTCGCCAAAAG (RV)
BA20	c.4446C>A	CAGAAATGGACACAGGTAAACCCTTCACCATCCTG (FW)
		CAGGATGGTGAAGGGTTTACCTGTGTCCATTTCTG (RV)
	c.4457C>T	AGGTCAACCCTTCACTATCCTGCAGGTGCAG (FW)
		CTGCACCTGCAGGATAGTGAAGGGTTGACCT (RV)
BA23	c.5088C>G	CCTTCGTCCCAGCCAGGTTTGTCCTTTATTTGAT (FW)
		ATCAAATAAAGGACAAACCTGGCTGGGACGAAGG (RV)
BA25	c.5367C>G	GTTTGATGTCCCCAGGACAGCCTATGTGGCT (FW)
		AGCCACATAGGCTGTCCTGGGGACATCAAAC (RV)
BA28	c.6339C>G	GCATGCTGTGGAACGTCATGGTGAGCATCATCA (FW)
		TGATGATGCTCACCATGACGTTCCACAGCATGC (RV)

Table S9. List of primers used for RT-PCR and Sanger sequencing.

Construct	Target	Sequence (5' - 3')	Amplicon length (bp)
**BA7**	Exon 7	TCTGAGATCTTGGGGAGGAA (FW)	748
	Exon 11	CTCCAGGTATTGATTGACCAG (RV)	
**BA12**	Exon 13	GCCTATCTGCAGGACATGGT (FW)	788
	Exon 17	CAAGGAAGTGGGGTTCCATA (RV)	
**BA15**	Exon 19	TCTTTGAACGTGAGCATCCA (FW)	503
	Exon 22	AATCACCACCTTGGCATCTC (RV)	
**BA16**	Exon 21	AGATGGAAGCCATGTTGGAG (FW)	440
	Exon 24	CGTGGTGGAGAAACCCTTAG (RV)	
**BA17**	Exon 23	TGCGCAAGATGAAAAACATC (FW)	357
	Exon 26	CCTCCGTGACCTTCAGAAAA (RV)	
**BA19**	Exon 26	GAAGGTCACGGAGGATTCTG (FW)	495
	Exon 29	CCTGGCTTATTCAGGAGGAC (RV)	
**BA20**	Exon 28	CCGGCTACCTTTGTGTTTTT (FW)	452
	Exon 31	ATGTTCCTGTCCGTCAGGTC (RV)	
**BA23**	Exon 32	GCAAATTCTGGGTCAATGAAC (FW)	578
	Exon 37	CCCAGCACTCACGGAATAAT (RV)	
**BA25**	Exon 36	TGAATTATTCCGTGAGTGCTG (FW)	517
	Exon 40	ATTGGGAGAGGAAGAAGTGG (RV)	
**BA28**	Exon 43	AAGATGCTCACTGGGGACAC (FW)	518
	Exon 47	GAATGGTGCCCATACATCG (RV)	
